# The evolutionarily conserved ESRE stress response network is activated by ROS and mitochondrial damage

**DOI:** 10.1186/s12915-020-00812-5

**Published:** 2020-06-29

**Authors:** Elissa Tjahjono, Aidan P. McAnena, Natalia V. Kirienko

**Affiliations:** Department of BioSciences, Rice University, 6100 Main St, MS140, Houston, TX 77005 USA

**Keywords:** Mitochondria, ESRE, UPR^mt^, Surveillance, Superoxide, Reductive stress

## Abstract

**Background:**

Mitochondrial dysfunction causes or contributes to a wide variety of pathologies, including neurodegenerative diseases, cancer, metabolic diseases, and aging. Cells actively surveil a number of mitochondrial readouts to ensure that cellular homeostasis is maintained.

**Results:**

In this article, we characterize the role of the ethanol and stress response element (ESRE) pathway in mitochondrial surveillance and show that it is robustly activated when the concentration of reactive oxygen species (ROS) in the cell increases. While experiments were mostly performed in *Caenorhabditis elegans*, we observed similar gene activation profile in human cell lines. The linear relationship between ROS and ESRE activation differentiates ESRE from known mitochondrial surveillance pathways, such as the mitochondrial unfolded protein response (UPR^mt^), which monitor mitochondrial protein import. The ability of the ESRE network to be activated by increased ROS allows the cell to respond to oxidative and reductive stresses. The ESRE network works in tandem with other mitochondrial surveillance mechanisms as well, in a fashion that suggests a partially redundant hierarchy. For example, mutation of the UPR^mt^ pathway results in earlier and more robust activation of the ESRE pathway. Interestingly, full expression of ATFS-1, a key transcription factor for the UPR^mt^, requires the presence of an ESRE motif in its promoter region.

**Conclusion:**

The ESRE pathway responds to mitochondrial damage by monitoring ROS levels. This response is conserved in humans. The ESRE pathway is activated earlier when other mitochondrial surveillance pathways are unavailable during mitochondrial crises, potentially to mitigate stress and restore health. However, the exact mechanisms of pathway activation and crosstalk remain to be elucidated. Ultimately, a better understanding of this network, and its role in the constellation of mitochondrial and cellular stress networks, will improve healthspan.

## Background

The survival of an organism critically depends on its ability to maintain its homeostasis in the face of constant disruptive forces. An inability to regain homeostasis inexorably leads to increased damage and ultimately death. The expression of transcription factors (TFs) that control tens to hundreds of genes coordinates the complex rearrangement of cellular resources to regain this homeostatic balance. Often, these TFs are constitutively expressed, poised to spring into action when needed. But because inappropriate expression can itself be pathological, they are generally kept in an inactive state until they are needed. Cellular surveillance programs that detect disruption of normal cellular processes license TF activity by triggering post-translational changes, such as re-localization, phosphorylation, or proteolytic cleavage. These patterns have been repeatedly observed across biological phyla for a variety of stresses, including heat shock [1], hypoxia [2], proteostatic disruption in the ER and mitochondria [3–5], and many others [6–8].

Mitochondria are bioenergetic hubs; synthetic factories for cholesterol, iron-sulfur groups, and heme; crucial sources of reactive oxygen species (ROS); and key regulators of iron and calcium homeostasis and programmed cell death pathways [9, 10]. Mitochondrial dysfunction plays a role in a staggering array of chronic conditions, including diabetes, cancer, and neurodegenerative diseases [9, 11]. Given the importance of these roles (and their extracellular origin), it is unsurprising that mitochondrial function is closely monitored.

Mitochondrial traits under surveillance include iron homeostasis [12, 13], lipid biosynthesis [14, 15], bioenergetics [16], and membrane potential and protein import [17–19]. To date, the most thoroughly studied mitochondrial surveillance pathway remains the mitochondrial unfolded protein response program (UPR^mt^) [20–24]. ATFS-1 in *Caenorhabditis elegans,* and its mammalian homolog ATF5, is the TF most directly responsible for regulating UPR^mt^ target genes. ATFS-1/ATF5 is normally trafficked to mitochondria where it is imported and rapidly degraded by matrix-resident proteases [20]. Mitochondrial stress compromises import efficiency, causing ATFS-1/ATF5 to accumulate in the cytoplasm instead. This allows a weaker, secondary signal in the protein to retarget the TF to the nucleus, where it drives expression of mitochondrial chaperones, like *hsp-6* and *hsp-60* [22]. There is some evidence that increased transcription of mitochondrial chaperones and other defense genes in the absence of stress promotes longevity [20], but these findings remain controversial [25].

Recently, the ESRE network has also been linked to mitochondrial surveillance by work from our lab and the Rea lab [16, 26]. The ESRE network was named for an 11-nucleotide motif (the *E*thanol and *S*tress *R*esponse *E*lement) found in the promoter region of a large number of genes that are activated in response to a variety of stresses, including ethanol and heat [27–30]. We have also shown that mitochondrial damage caused by removal of iron by either pyoverdine (a bacterial siderophore produced by the multispecies pathogen *Pseudomonas aeruginosa*) or 1,10-phenanthroline (a synthetic iron-chelating compound) triggers expression of ESRE-containing genes [26]. Based on observations that pyoverdine and phenanthroline damage mitochondria [10, 12, 31], we predicted that the ESRE network surveils mitochondria and responds to their damage. Indeed, exposure to mitochondrial poisons rotenone and antimycin A elevated ESRE gene transcripts [26].

Importantly, the ESRE network shows strong evolutionary conservation. Drosophila and mammals both possess orthologs of *C. elegans* ESRE genes that, in most cases, retain the ESRE motif and respond to ESRE-activating conditions, including heat, ethanol, and mitochondrial damage [26, 28, 30]. The retention of this network and particularly the ESRE motif, which is located in intergenic regions more prone to genetic drift than their coding counterparts, across a striking evolutionary distance indicates an important role in cellular health.

In this paper, we demonstrate that ESRE reporter expression correlates significantly with ROS levels. Interestingly, the addition of N-acetyl cysteine (NAC), a canonical antioxidant, exacerbates ROS production and ESRE activation, particularly during malfunction of complex I of the mitochondrial electron transport chain (ETC), conditions that are associated with reductive stress. Our data indicate that the ESRE network may be the first recognized system for surveilling reductive stress. We also show substantial interaction amongst several mitochondrial surveillance pathways. For example, we show that both the UPR^mt^ and a *C. elegans* mitochondrial surveillance pathway controlled by a DLK-1/MAPKKK, SEK-3/MAPKK, and PMK-3/MAPK cascade discovered by the Rea lab that we refer to as MAPK^mt^ repress activation of the ESRE network in *C. elegans*. We also show that the promoter region of the *atfs-1* gene includes an ESRE element that is required for its full expression.

## Results

### Mitochondrial surveillance pathways exhibit partial redundancy

**Fig. 1:**
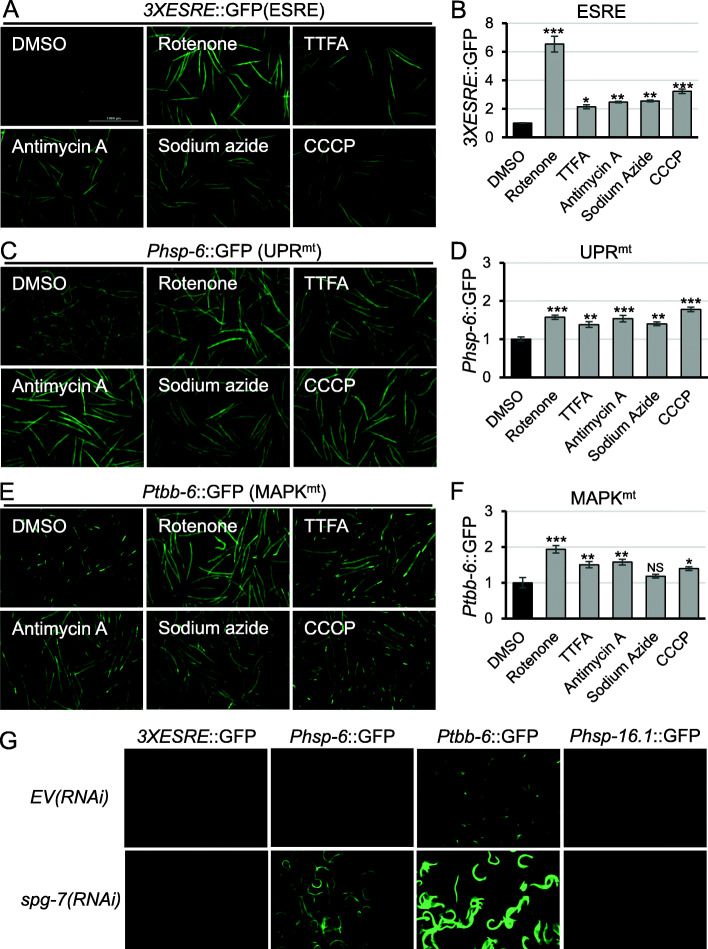
Multiple mitochondrial pathways were activated by mitochondrial-damaging agents. **a**, **c**, **e** Fluorescent images and **b**, **d**, **f** quantification of GFP fluorescence of *C. elegans* carrying **a**, **b***3XESRE*::GFP; **c**, **d***Phsp-6*::GFP; or **e**, **f***Ptbb-6*::GFP reporters that were treated for 10 h with 50 μM rotenone, 3 mM TTFA, 50 μM antimycin A, 10 mM sodium azide, 20 μM CCCP, or vehicle (DMSO). **g** Fluorescent images of worms with *3XESRE*::GFP (most left), *Phsp-6*::GFP (second from the left), *Ptbb-6*::GFP (second from the right), or *Phsp-16.1*::GFP (most right) reared on *E. coli* containing empty vector (*EV*) (top) or *spg-7(RNAi)* (bottom). Representative images are shown; three biological replicates with ~ 400 worms/replicate were analyzed. Error bars represent SEM. *p* values were determined from one-way ANOVA, followed by Dunnett’s test. All fold changes were normalized to DMSO control. NS not significant, *p < 0.05, **p < 0.01, ***p < 0.001. See Fig. S1 in Additional File 1 for quantification for **g**

To test the variety of mitochondrial damage that can activate the ESRE network, a *C. elegans* strain carrying a GFP reporter driven by three tandem repeats of the minimal 11-nt ESRE consensus (*3XESRE*::GFP) [28] was exposed to a panel of mitochondrial poisons including rotenone (complex I inhibitor), TTFA (thenoyltrifluoroacetone, complex II inhibitor), antimycin A (complex III inhibitor), sodium azide (complex IV inhibitor), and carbonyl cyanide 3-chlorophenylhydrazone (CCCP, a protonophore that dissipates the electrochemical gradient across the inner mitochondrial membrane) (Fig. 1a, b). Strains carrying *Phsp-6*::GFP [20] or *Ptbb-6*::GFP [16] (reporters for UPR^mt^ or MAPK^mt^, respectively) were tested in parallel (Fig. 1c–f). With the exception of sodium azide (which did not activate the MAPK^mt^ pathway, but did activate the others), each drug significantly activated all three pathways (Fig. 1a, c, e, quantifications in Fig. 1b, d, f). Activation of endogenous ESRE genes measured via qRT-PCR following rotenone treatment confirmed reporter results (Additional File 1**:** Fig. S1).

Interestingly, *spg-7/SPG7*(*RNAi*), which is a strong activator of both the UPR^mt^ [21] and the MAPK^mt^ pathways [16], did not trigger expression of the *3XESRE*::GFP reporter in otherwise healthy worms (Fig. 1g, quantification in Additional File 1: Fig. S2A). SPG-7 is the *C. elegans* ortholog of the human Paraplegin protein, an inner mitochondrial membrane-localized protease involved in mitochondrial protein quality control [32, 33]. This was not an artifact caused by the minimal promoter construct used; *spg-7/SPG7(RNAi)* also failed to activate a *Phsp-16.1*::GFP reporter (Fig. 1g, quantification in Additional File 1: Fig. S2A). This construct contains a native, full-length promoter; responds to ESRE stimuli (Additional File 1: Fig. S2B); and contains two ESRE motifs [27]. In addition, the *3XESRE*::GFP reporter was robustly activated by other mitochondrial toxins.

To determine the breadth of ESRE surveillance, we tested whether other mitochondrial perturbations activated the *3XESRE*::GFP reporter. RNAi was used to knock down several mitochondrial components, including *cco-1*/*COX5B* (cytochrome c oxidase subunit 5B), *mrps-5*/*MRPS5* (mitochondrial ribosomal protein S5), *tomm-20*/*TOMM20* (translocase of outer mitochondrial membrane 20), and *tomm-22*/*TOMM22* (translocase of outer mitochondrial membrane 22). In each case, the ESRE reporter remained dim (Additional File 1: Fig. S3A), further differentiating this surveillance network from UPR^mt^ and MAPK^mt^ which both showed strong response to each knockdown (Additional File 1: Fig. S3B, C).

To ensure that ESRE pathway expression was being triggered by mitochondrial insults rather than general cell stress, worms carrying the *3XESRE*::GFP reporter were treated with the proteasomal inhibitor bortezomib, which induces proteasomal stress, or the N-glycosylation inhibitor tunicamycin, which induces ER stress [34]. Although neither compound activated *3XESRE::*GFP expression during a 20-h course of treatment, they did activate *Prpt-3*::GFP [35] and *Phsp-4*::GFP [34] control reporters, showing that they were behaving as expected (Additional File 1: Fig. S4). As ESRE activation usually occurs within 6–10 h, it is unlikely that this time frame was insufficient.

We also tested each of the three reporters with a variety of abiotic stresses that activate the ESRE stress response network [27–31], including ethanol, sodium selenite, phenanthroline (an iron chelator), and heat shock (Additional File 2: Table S1). Generally, significant overlap in activation was observed amongst ESRE, UPR^mt^, and MAPK^mt^, but disparities were easily identified, suggesting that these pathways likely have both shared and independent functions.

### Reductive stress induces ESRE activation

**Fig. 2:**
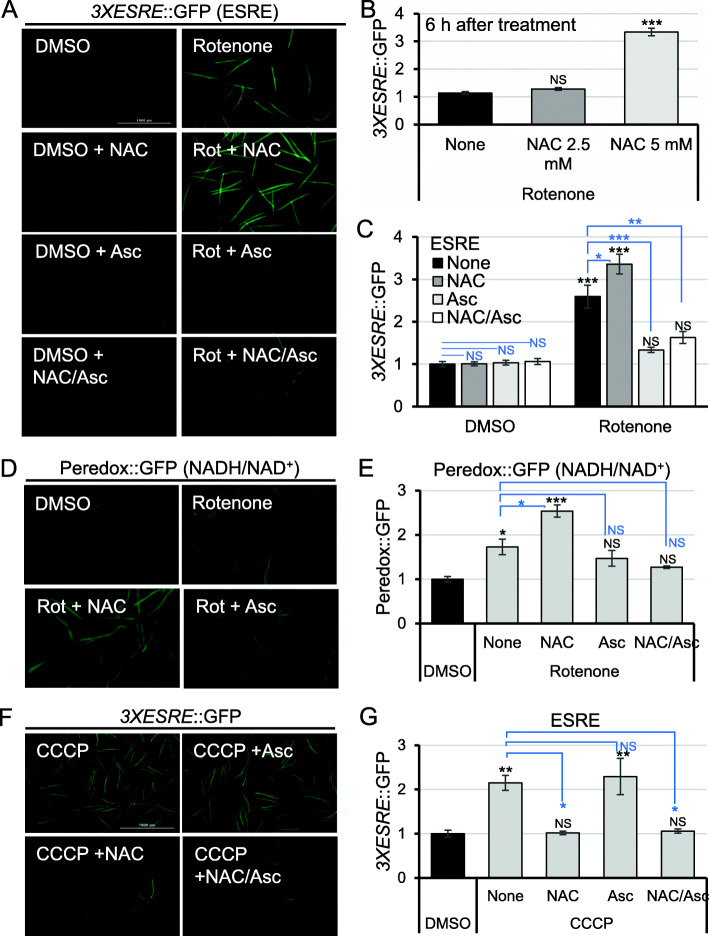
The combination of rotenone and NAC enhanced ESRE expression. **a** Fluorescent images of *C. elegans* strains with *3XESRE*::GFP reporter treated with vehicle (DMSO) (left) or 50 μM rotenone (right) with or without 25 mM ascorbate (Asc), 2.5 mM NAC, or ascorbate and NAC combination (NAC/Asc) for 8 h. **b** Quantification of GFP fluorescence for *3XESRE*::GFP reporter after 6-h treatment with rotenone with or without 2.5 mM NAC or 5 mM NAC. **c** Quantification of GFP fluorescence for **a**. **d** Fluorescent images and **e** quantification of GFP fluorescence of *C. elegans* carrying Peredox::GFP (NADH/NAD^+^) reporter treated with vehicle (DMSO), rotenone, or rotenone with 2.5 mM NAC and/or 25 mM ascorbate for 10 h. **f** Fluorescent images and **g** quantification of GFP fluorescence of *C. elegans* with *3XESRE*::GFP reporter following treatment with CCCP alone or in combination with 2.5 mM NAC, 25 mM ascorbate, or NAC and ascorbate (NAC/Asc) for 10 h. Representative images are shown. Three biological replicates with ~ 400 worms/replicate were analyzed. Error bars represent SEM. *p* values were determined from one-way ANOVA, followed by Dunnett’s test. GFP values were normalized to DMSO. NS not significant, *p < 0.05, **p < 0.01, ***p < 0.001

Rotenone is the most potent activator of the minimal *3XESRE*::GFP reporter that we have observed. Consistent with earlier findings for ESRE activation [28, 36], *3XESRE*::GFP expression became noticeable after approximately 8 h of treatment and stayed high until death. Since complex I poisoning by rotenone is known to trigger release of reactive oxygen species (ROS) into mitochondria [37], we hypothesized that ROS production may be involved in ESRE pathway activation. To test this prediction, two antioxidants, N-acetyl cysteine (NAC, a precursor for glutathione [38]) and ascorbate (vitamin C [39, 40]), were added during rotenone treatment to determine if scavenging ROS would limit ESRE activation. The addition of ascorbate to *3XESRE*::GFP worms treated with rotenone had the anticipated effect and reduced GFP fluorescence (Fig. 2a). Surprisingly however, the addition of 5 mM NAC had the opposite effect and increased, rather than reduced, ESRE activation (Fig. 2b). GFP expression was detectable earlier (6 h instead of 8 h), and the intensity was three times higher than with rotenone alone. Reducing the NAC concentration by half, to 2.5 mM, still increased GFP expression compared to rotenone only, but allowed direct comparison of treatments at 8 h (Fig. 2a, c). In contrast, the addition of NAC to rotenone had no effect on the activation of the UPR^mt^ or MAPK^mt^ pathways (Additional File 1: Fig. S5). It is unlikely that exacerbation of ESRE activation was due to NAC affecting fluorescence; NAC alone (in DMSO control) did not cause reporter expression for any of the pathways (Fig. 2a, c, Additional File 1: Fig. S5). Adding ascorbate to *3XESRE*::GFP worms treated with rotenone and NAC still attenuated ESRE activation (Fig. 2a, c).

The explanation for this was not immediately obvious. However, searching the literature, led to a potential explanation. Rotenone poisoning of complex I in mitochondria prevents transfer of electrons from NADH to coenzyme Q_10_, leading to an increase in the NADH/NAD^+^ ratio. NAC, which is directly reductive and can be a substrate for glutathione synthesis (another reducing substance), can further increase the available pool of reducing equivalents. These equivalents can deplete the cell of ROS, inducing a state of reductive stress [41–43]. This state can substantially alter the redox chemistry of the cell and can counterintuitively generate ROS by the direct transfer of electrons to O_2_ ([44], see also the “Discussion” section).

To test this hypothesis, the NADH/NAD^+^ ratio was assessed in worms treated with rotenone, NAC, or both using the Peredox reporter [31, 45]. Fluorescence in this reporter is driven by an NADH-dependent conformational change, providing a semi-quantitative readout of the redox status of the cell. Consistent with our hypothesis, rotenone-triggered Peredox fluorescence was significantly increased when NAC was added (Fig. 2d, e). To confirm that this activity was specific to complex I-related damage, we substituted rotenone with CCCP. In direct contrast to its effect on rotenone treatment, the addition of NAC to CCCP largely eliminated *3XESRE*::GFP fluorescence (Fig. 2f, g). Interestingly, the addition of ascorbate had no apparent effect on ESRE activation induced by CCCP. This suggests that the function of different antioxidants depends on biochemical events within the cell and that they should not be considered interchangeable.

### ESRE network activation correlates with ROS level

As noted above, the increased NADH/NAD^+^ ratio and increased ESRE activation after treatment with NAC suggest that the ESRE network may be sensing reductive stress. If this is the case, there should be increased superoxide and/or hydrogen peroxide in *C. elegans* treated with rotenone and NAC. To test whether treatment with rotenone and NAC generated ROS, worms were stained with dihydroethidium (DHE), a redox-sensitive dye widely used for the detection of superoxide. The reaction between DHE and superoxide results in the formation of a fluorescent product 2-hydroxyethidium (2-OH-E+) that can be easily quantified [46, 47].

However, there are concerns regarding the interpretation of DHE fluorescence as a specific indicator of superoxide, since the reaction of DHE with different ROS produces several oxidative products that are difficult to discriminate [46–50]. For example, DHE has been shown to be oxidized to ethidium (E+) by H_2_O_2_, which is different from 2-OH-E+ [46, 47] but with overlapping spectra that are exceptionally difficult to deconvolve [51]. Further complicating matters, superoxide is rapidly converted into hydrogen peroxide (H_2_O_2_), either spontaneously or by superoxide dismutases (SODs) [52], and there is growing evidence that excess hydrogen peroxide can trigger the production of superoxide [53–56]. Consequently, it is difficult to unambiguously identify the ROS present in the worms. For these reasons, the general term ROS will be used to interpret DHE results.

**Fig. 3:**
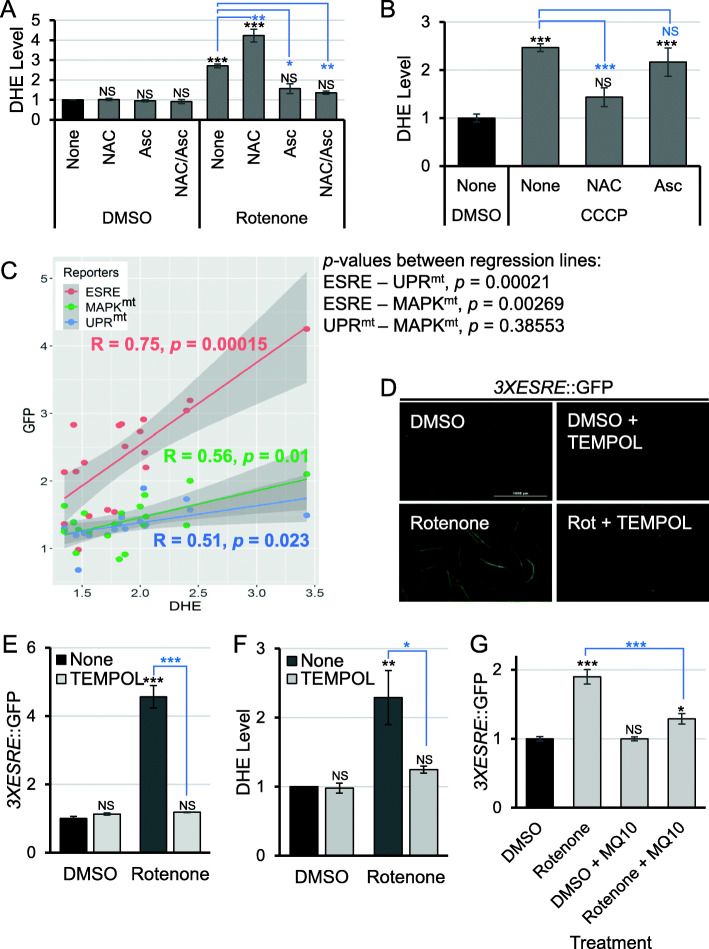
ESRE reporter activation correlates with DHE fluorescence**. a** Cellular ROS content was assessed based on DHE fluorescence measurement after treatment with vehicle (DMSO) or rotenone with or without 2.5 mM NAC, 25 mM ascorbate (Asc), or ascorbate and NAC combination (NAC/Asc) for 10 h. **b** Quantification of DHE fluorescence after the treatment with vehicle (DMSO) or CCCP with or without 2.5 mM NAC or 25 mM ascorbate (Asc) for 10 h. **c** Correlation of ROS level (DHE fluorescence) with reporters’ expression. **d** Fluorescent images and **e** quantification of GFP fluorescence of worms carrying *3XESRE*::GFP reporter after treatment with vehicle (DMSO) (top) or rotenone (bottom) with or without 5 mM TEMPOL for 10 h. **f** Quantification of DHE fluorescence under same treatment conditions as in **d**, **e**. **g** Quantification of GFP fluorescence for *3XESRE*::GFP reporter after treatment with vehicle (DMSO) or rotenone with or without 10 μM mitoquinol (MQ10) for 10 h. Representative images for **d** are shown. Three biological replicates with ~ 400 worms/replicate were analyzed. Error bars represent SEM. *p* values were determined from one-way ANOVA, followed by Dunnett’s test. All fold changes were normalized to DMSO. NS not significant, *p < 0.05, **p < 0.01, ***p < 0.001

Consistent with our predictions, the addition of NAC to rotenone caused a surge of DHE fluorescence (Fig. 3a). In contrast, adding ascorbate reduced ESRE fluorescence and attenuated DHE fluorescence after rotenone treatment, with or without NAC. Both observations corroborate our hypothesis. Since the pattern of ESRE activation using CCCP differed from rotenone, DHE staining was also performed after exposure to CCCP in the presence or absence of either NAC or ascorbate. In this case, NAC, but not ascorbate, significantly reduced DHE signal (Fig. 3b), again matching ESRE fluorescence. Finally, we took advantage of the fact that *spg-7(RNAi)* did not activate the ESRE reporter to use this as a negative control. We measured basal DHE fluorescence of *spg-7(RNAi)* worms and saw that they were indistinguishable from vector controls (Additional File 1: Fig. S6).

Several mitochondria-damaging conditions induced ROS production and activated all three mitochondrial surveillance pathways. For this reason, DHE fluorescence and reporter expression were measured under a variety of conditions to determine whether a broad correlation exists (Fig. 3c). Across the conditions tested, ESRE expression strongly and significantly correlated with ROS levels as detected by DHE staining; conditions that increased ROS concentration increased ESRE expression to nearly identical levels (e.g., direct linear correlation: ~ 3-fold increase in ROS resulted in ~ 3.5-fold increase in ESRE reporter). Interestingly, UPR^mt^ and MAPK^mt^ reporter upregulation only moderately correlated with ROS levels (e.g., 3.5-fold increase in ROS resulted in 2- and 1.5-fold increase in reporter expression, respectively). Since the pattern of ESRE expression correlated with DHE signals, we hypothesized that the ESRE network may be surveilling reductive stress by monitoring ROS levels.

To test whether the ESRE pathway is generally activated by ROS-producing compounds, we tested whether juglone (a well-known producer of ROS) could also activate the ESRE reporter. Consistent with our prior experiments, we observed increases in DHE staining and ESRE gene expression following juglone exposure (Additional File 1: Fig. S7). *gst-4*, a SKN-1-dependent gene that has been well-characterized to respond to juglone [57] was used as a positive control.

In a larger context, SKN-1 is generally considered to be a key regulator of the response to oxidative damage and toxins [58–60]. On this basis, we tested whether the *Pgst-4*::GFP reporter would respond to the conditions that activated the *3XESRE*::GFP. Only juglone activated the *Pgst-4*::GFP reporter (Additional File 1: Fig. S8). Two explanations for these observations are possible: first, the level of damage inflicted by these compounds could be lower than the threshold for activation of *Pgst-4*::GFP. Second, it remains possible that the compounds are generating ROS of a particular type or in a particular location that does not activate the SKN-1 response. In either case, these data argue that the ESRE network exhibits selectivity.

To further test the relationship between ROS and ESRE, we used 4-hydroxy-TEMPO (TEMPOL, 4-hydroxy-2,2,6,6-tetramethyl-1-piperidinyloxy), a cyclic piperidine nitroxide that mimics the function of SOD enzymes [61–63]. It does this by preferentially scavenging superoxide, although it can also catalyze the metabolism of other ROS [64], predominantly from the cytoplasm. TEMPOL reduced both *3XESRE*::GFP fluorescence (Fig. 3d, e) and DHE staining (Fig. 3f) of rotenone-treated worms. A similar phenomenon (decreased *3XESRE*::GFP fluorescence after rotenone treatment) was seen when mitoquinol (MitoQ or MQ10) was used instead (Fig. 3g). MQ10, a ubiquinol derivative targeted to mitochondria, acts as a mitochondria-specific scavenger for ROS [65]. These results may indicate that ROS in either compartment is sufficient for ESRE activation or that ROS (or its effects) can travel between the compartments. For example, hydrogen peroxide freely travels through membranes while mitochondrially generated superoxide can travel through the voltage-dependent anion channel [66].

As an alternative method to attempt to distinguish the location of ROS that induces ESRE, we obtained knockout mutants for several SOD genes and quantified the mRNA levels of ESRE target genes in these mutants. The loss of both mitochondrial superoxide dismutases (*sod-2;sod-3*) or, to a some extent, *sod-2* alone, increased basal expression of target genes (Additional File 1: Fig. S9). The loss of cytoplasmic SODs (*sod-1* or *sod-5*) or the extracellular SOD (*sod-4*) had less effect. These results are consistent with, but do not prove, our hypothesis that the ESRE pathway is induced by mitochondrial superoxide. However, it remains unclear whether this occurs directly, indirectly through a mediator, or through the modulation of cytoplasmic ROS level.

### ESRE responds to exogenous H_2_O_2_ but does not correlate with endogenous peroxide levels

In a further attempt to investigate whether it was superoxide or the resulting hydrogen peroxide that induced ESRE gene expression, worms were treated with exogenous H_2_O_2_ and *3XESRE*::GFP levels were measured. Exogenous H_2_O_2_ triggered expression of both ESRE and UPR^mt^ reporters (Additional File 1: Fig. S10A). However, adding H_2_O_2_ increased fluorescence of both DHE and Amplex Red (which exclusively measures cellular H_2_O_2_) [67] (Additional File 1: Fig. S10B, C).

To attempt to gain insight into the sensitivity of DHE to O_2_^−^ and H_2_O_2_ under our assay conditions, worms were exposed to a gradient of exogenous H_2_O_2_ concentrations and then stained with Amplex Red or DHE. As expected, the correlation of Amplex Red fluorescence with H_2_O_2_ was almost perfectly directly linear, with a 1:1 ratio (Additional File 1: Fig. S11A). In contrast, the relationship between DHE and H_2_O_2_ was clearly non-linear (Additional File 1: Fig. S11B). Instead, DHE fluorescence remained low as the concentration increased, and then began to increase linearly. Two explanations for this phenomenon suggest themselves. First, it may indicate the saturation detoxification process for H_2_O_2_, and once saturated, the DHE fluorescence begins to increase. Alternatively, it is possible that H_2_O_2_-mediated superoxide production only starts once peroxide reaches a threshold concentration.

We compared *3XESRE*::GFP expression, which was also measured in the same H_2_O_2_ gradient, to staining with Amplex Red and DHE. *3XESRE*::GFP fluorescence correlated much more strongly with DHE fluorescence than with Amplex Red staining (Additional File 1: Fig. S11C, D).

Finally, the level of H_2_O_2_ was measured with Amplex Red after worms were treated with the same panel of mitotoxins as were used earlier in this report (Additional File 1: Fig. S10D). Of the five compounds, only TTFA was different from DMSO, suggesting that the ROS produced by the compounds were not H_2_O_2_. We also determined correlation coefficients for each of the three mitochondrial surveillance reporters with Amplex Red fluorescence. In contrast to DHE fluorescence, no correlation was found between Amplex Red fluorescence and any of the mitochondrial surveillance pathways (compare Additional File 1: Fig. S10E and Fig. 3c). Combined, these data argue that the ESRE network is more likely to be responding to superoxide than hydrogen peroxide.

### UPR^mt^ and the MAPK^mt^ pathways restrict ESRE expression by limiting ROS production

To test for interactions between mitochondrial surveillance pathways, key regulators for each pathway (when known) were genetically disrupted. First, *atfs-1*/ATF5 and *pmk-3*/MAPK were knocked down via RNAi, and (due to the TF for the ESRE reporter remaining unidentified) expression was assessed in RNAi-fed strains under basal and induction conditions.

**Fig. 4:**
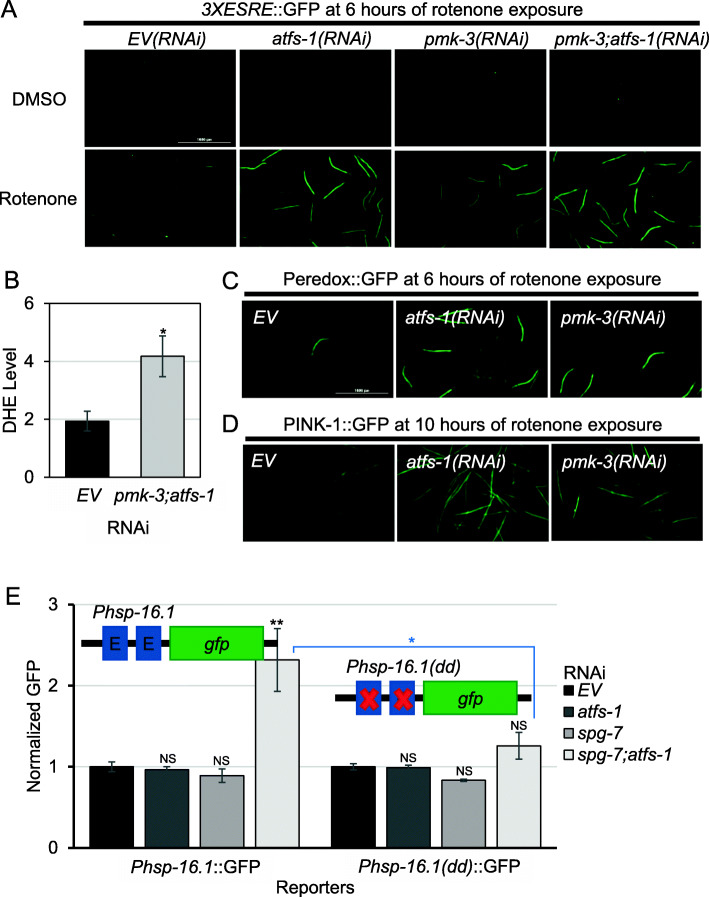
The loss of UPR^mt^ and MAPK^mt^ pathways caused early activation of the ESRE reporter. **a** Fluorescent images of worms expressing *3XESRE*::GFP reporter reared on *E. coli* expressing empty vector (*EV*), *atfs-1(RNAi)*, *pmk-3(RNAi)*, or *pmk-3(RNAi); atfs-1(RNAi)*. Worms were treated with 50 μM rotenone or vehicle (DMSO). Images were taken at 6 h after treatment. See Fig. S12 in Additional File 1 for quantification. **b** Quantification of DHE fluorescence of N2 worms reared on *E. coli* expressing empty vector (*EV*) or *pmk-3(RNAi); atfs-1(RNAi)*. Worms were treated with 50 μM rotenone for 10 h. Fold changes were normalized to *EV* on DMSO. **c** Fluorescent images of Peredox::GFP reporter reared on *E. coli* expressing *EV*, *atfs-1(RNAi)*, or *pmk-3(RNAi)*. **d** Fluorescent images of PINK-1::GFP reporter reared on *E. coli* expressing empty vector (EV), *atfs-1(RNAi)*, or *pmk-3(RNAi)*. **e** Quantification of GFP fluorescence of ESRE native reporters with intact (*Phsp-16.1*::GFP) or removed (*Phsp-16.1(dd)*::GFP) ESRE motifs. Worms were reared on *E. coli* expressing empty vector (EV), *atfs-1(RNAi)*, *spg-7(RNAi)*, or *spg-7(RNAi); atfs-1(RNAi)* for 2 days. GFP values were normalized to *EV*. Representative images are shown; three biological replicates with ~ 400 worms/replicate were analyzed. Error bars represent SEM. *p* values were determined from **b** Student’s *t* test or **e** one-way ANOVA followed by Dunnett’s test. NS not significant, *p < 0.05, **p < 0.01

Under basal conditions, ESRE expression was unaffected by the presence or absence of functional *atfs-1*/ATF5, *pmk-3*/MAPK, or both (Fig. 4a). In contrast, ESRE activation was significantly affected when these pathways were compromised during mitochondrial stress. For example, the *3XESRE*::GFP reporter was activated earlier (4 h after treatment in *atfs-1/ATF5(RNAi*) mutant, 5–6 h in *pmk-3/MAPK(RNAi)*, compared to 8 h for vector) and fluorescence levels were higher (Fig. 4a, quantifications in Additional File 1: Fig. S12A). Disrupting both UPR^mt^ and MAPK^mt^ created an additive effect. On first glance, the lack of an apparent change in ESRE activation under basal conditions but increased activation under stress may appear inconsistent. However, inactivation of the UPR^mt^ and MAPK^mt^ pathways did result in higher levels of ROS (as measured by DHE staining, Fig. 4b) after stress. This was consistent with observations that both NADH accumulation and mitophagic activation occurred more rapidly after rotenone exposure if the UPR^mt^ or MAPK^mt^ pathways were disrupted (Fig. 4c, d).

Next, *spg-7/SPG7* was knocked down via RNAi alone or in combination with *atfs-1/*ATF5. *spg-7* disruption had no apparent effect, alone or in combination with *atfs-1*/ATF5*(RNAi)*, on the expression of the minimal ESRE reporter (Additional File 1: Fig. S12B). As this conflicted with a report from the Rea lab, where the double knockdown induced ESRE gene expression [16], we tested a more biologically accurate reporter construct, the *Phsp-16.1*::GFP reporter. The promoter region of *hsp-16.1* is more complex and includes multiple regulatory elements, including two ESRE motifs [68]. Unlike the *3XESRE*::GFP reporter, the *Phsp-16.1*::GFP reporter was activated by *spg-7(RNAi); atfs-1(RNAi)* in an ESRE-dependent fashion (Fig. 4e).

### Intricate genetic interactions exist between the UPR^mt^ and the MAPK^mt^ pathways

**Fig. 5:**
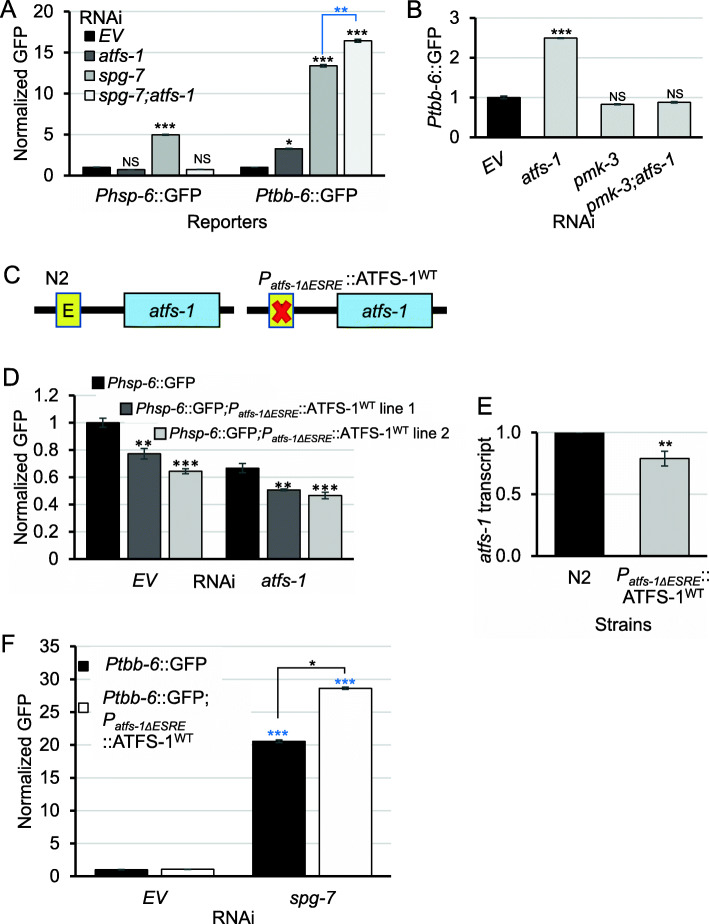
ESRE motif in *atfs-1* is necessary for full *atfs-1* expression. **a** Quantification of GFP fluorescence of *C. elegans* carrying *Phsp-6*::GFP (left) or *Ptbb-6*::GFP (right) reporters reared on *E. coli* expressing empty vector (*EV*), *atfs-1(RNAi)*, *spg-7(RNAi)*, or *spg-7(RNAi); atfs-1(RNAi)*. **b** Quantification of GFP fluorescence of *Ptbb-6*::GFP reporter reared on *E. coli* expressing empty vector (*EV*), *atfs-1(RNAi)*, *pmk-3(RNAi)*, or *pmk-3(RNAi); atfs-1(RNAi)*. **c** Schematic of a mutant in which the ESRE motif located in the *atfs-1* promoter was removed (*Patfs-1ΔESRE::*ATFS-1^WT^, right), compared to wild type (N2, left). **d** Quantification of GFP fluorescence of *Phsp-6*::GFP in two lines of *Phsp-6*::GFP;*Patfs-1ΔESRE*::ATFS-1^WT^ reporters reared on *E. coli* expressing empty vector (*EV*) or *atfs-1(RNAi)*. Fold changes were normalized to *Phsp-6*::GFP reared on *EV*. **e** Expression of *atfs-1* gene in N2 wild type or *Patfs-1ΔESRE*::ATFS-1^WT^ worms on basal level. Fold change was normalized to N2. **f** Quantification of GFP fluorescence of *Ptbb-6*::GFP or *Ptbb-6*::GFP;*Patfs-1ΔESRE*::ATFS-1^WT^ reporters reared on *E. coli* expressing empty vector (*EV*) or *spg-7(RNAi)*. Fold changes were normalized to *Ptbb-6*::GFP reared on *EV*. Three biological replicates with ~ 1000 worms/replicate were analyzed. All fold changes were normalized to *EV*. Error bars represent SEM. *p* values were derived from **a**, **b**, **d**, **f** one-way ANOVA, followed by Dunnett’s test, or **e***t* test. NS not significant, *p < 0.05, **p < 0.01, ***p < 0.001

Previous work claimed that expression of the MAPK^mt^ pathway was enhanced by loss of *atfs-1/ATF5* during stress, but that the basal level was unaffected [16]. We attempted to recapitulate these data to gain a finer understanding of the interrelationship of these genetic pathways. In contrast to earlier observations, we saw that *atfs-1(RNAi)* increased *Ptbb-6*::GFP expression under non-stressed conditions (Fig. 5a) and that activation was further increased by stress induction with *spg-7(RNAi)*. Combining *spg-7(RNAi)* with *atfs-1/ATF5(RNAi)* further increased activation of *Ptbb-6*::GFP, compared to *spg-7(RNAi)* alone, as was previously observed [16]. qRT-PCR measuring endogenous expression of MAPK^mt^ target genes under *atfs-1(RNAi)* condition in the absence of stress confirmed basal upregulation (Additional File 1: Fig. S13A). These data suggest that the UPR^mt^ normally serves to restrict MAPK^mt^ pathway activity, either directly or by limiting the accumulation of MAPK^mt^ pathway activators. We also showed that this repression was dependent on the MAPK^mt^ regulatory network, as *pmk-3(RNAi)* was epistatic to *atfs-1(RNAi)*. These observations were made for *Ptbb-6*::GFP (Fig. 5b) and for native MAPK^mt^ targets (Additional File 1: Fig. S13B). We verified that this did not result from an off-target RNAi result by testing a CRISPR-generated deletion in *atfs-1/*ATF5 (*atfs-1(cmh15)*) [69], which showed essentially the same results (Additional File 1: Fig. S13C).

During our investigation of the interrelationship of these pathways, we identified a full-length, perfect consensus match ESRE motif (TCTGCGTCTCT) in the promoter of *atfs-1*, located at − 87 to − 73 bp upstream of the transcriptional start site. Based on our previous experience, the presence of a single ESRE motif can be sufficient to mediate regulation of the gene, suggesting that *atfs-1* may be a part of the ESRE network. To test the importance of the motif in *atfs-1* expression, CRISPR/Cas9 was used to generate a precise deletion, removing only these 11 nucleotides (*Patfs-1ΔESRE*::ATFS-1^WT^*)* (Fig. 5c). Crossing this mutant with the *Phsp-6*::GFP and *Ptbb-6*::GFP reporter strains allowed us to observe that the expression of the *Phsp-6*::GFP reporter was decreased after removal of the ESRE site (Fig. 5d) and that *atfs-1(RNAi)* further diminished fluorescence (Fig. 5d). mRNA levels of *atfs-1* and UPR^mt^ targets were also decreased in *Patfs-1ΔESRE*::ATFS-1^WT^ worms (Fig. 5e, Additional File 1: Fig. S14A). Meanwhile, we observed a moderate increase in the expression of the MAPK^mt^ reporter, *Ptbb-6*::GFP, and endogenous MAPK^mt^ target expression when *Patfs-1ΔESRE*::ATFS-1^WT^ worms were reared on *spg-7(RNAi)* (Fig. 5f, Additional File 1: Fig. S14B). These results indicated the importance of the ESRE motif in the *atfs-1* promoter for normal ATFS-1 expression and further indicate the presence of extensive crosstalk amongst mitochondrial surveillance pathways.

### Human ESRE genes were upregulated by ROS

**Fig. 6:**
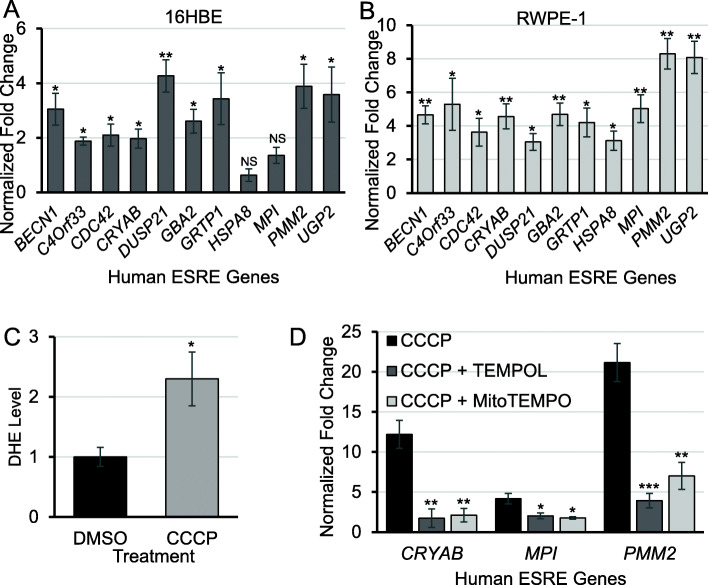
Human ESRE genes were upregulated by CCCP. Expression of human orthologs of ESRE genes in **a** bronchial epithelial cells (16HBE) and **b** human prostate epithelial cells (RWPE-1) after treatment with 20 μM CCCP for 16 h. **c** Quantification of DHE fluorescence of RWPE-1 cells after treatment with 20 μM CCCP for 16 h. **d** Expression of human orthologs of ESRE genes in RWPE-1 cells after treatment with 20 μM CCCP alone or in combination with 0.5 mM TEMPOL or 2.5 μM MitoTEMPO for 16 h. Expression levels were normalized to vehicle (DMSO) control. Three biological replicates were analyzed. Error bars represent SEM. *p* values were determined from **a**–**c***t* test and **d** one-way ANOVA, followed by Dunnett’s test. NS not significant, *p < 0.05, **p < 0.01, ***p < 0.001

Considering the high degree of conservation of the ESRE motif in humans [28], we sought to determine whether ROS levels influenced the expression of human orthologs of *C. elegans* ESRE genes. As noted previously, human orthologs of *C. elegans* ESRE genes frequently retain both the motif and responsiveness to ESRE-activating stimuli, despite the evolutionary distance [26, 28]. We used bronchial epithelial cells (16HBE) and human prostate epithelial cells (RWPE-1) to measure transcription for a panel of 10 human ESRE genes [26] via quantitative RT-PCR after exposure to CCCP (Fig. 6a, b). In general, the expression of ESRE genes in human cells followed the pattern observed in *C. elegans*. DHE staining verified that CCCP induced ROS production in RWPE-1 cells (Fig. 6c). Next, we measured the expression of the three most strongly induced ESRE genes in RWPE-1 cells treated with CCCP alone or in combination with TEMPOL or MitoTEMPO (a mitochondrial-targeted TEMPOL [54]). Similar to *C. elegans*, addition of either antioxidant decreased ESRE gene expression following CCCP induction (Fig. 6d). Combined, these data argue that ESRE gene expression is likely to respond to ROS concentration in both humans and *C. elegans* and that this may be the first stress response system known to be activated by reductive stress.

## Discussion

Metazoan life requires an elegant biochemical dance that can only take place on the backdrop of a homeostatic environment. Perturbation inevitably results in death unless healthy function can be restored. Historically, the mechanisms used to regain a homeostatic environment have been considered innate immunity (if the stressor was a pathogen) or stress responses (if the stressor was abiotic). From the perspective of the cell, this is a distinction without a difference; eIF2α phosphorylation decreases translation regardless of whether it occurs due to nutrient deprivation, heat shock, iron deficiency, or exposure to *Pseudomonas aeruginosa* Exotoxin A [70, 71].

Given these observations, it is unsurprising that complex surveillance systems exist to monitor most of the essential functions of cells (sometimes called cSADDs) [72], and it seems reasonable to assume that systems that are more essential for survival or reproduction or that are more metabolically active may have more robust or redundant surveillance pathways. The centrality of mitochondria to cellular energy production, their role in calcium and iron homeostasis, their ability to trigger apoptosis, and their extracellular origins all suggest that mitochondria are likely to be heavily monitored for aberrant function. Consequently, the most surprising thing about the recent proliferation of identified mitochondrial surveillance systems is not that there are so many systems [15, 16, 19–21, 26] but rather that it took so long for them to begin to be recognized. Since mitochondrial dysfunction is increasingly recognized as a causative factor in many, if not most, chronic diseases, it is critical that we obtain a better understanding of these quality control mechanisms.

Until recently, almost nothing was known about any of these pathways except for UPR^mt^ [20, 21, 23] and the PINK1/Parkin axis of mitophagy [73], both of which essentially depend on mitochondrial protein import as a readout of organellar health. As others have been discovered, additional indicators, such as changes in mitochondrial membrane potential (mitoCPR, ESRE), lipid metabolism (MCSR), and ROS (ESRE), have been shown to allow rapid activation of mitochondrial surveillance pathways [15, 19, 26].

Interestingly, there appears to be considerable redundancy and interplay amongst some of these pathways. The three pathways studied here (the UPR^mt^, MAPK^mt^, and ESRE networks) exhibited substantial, but incomplete, overlap in response to ETC inhibitors, an ionophore that dissipated the mitochondrial membrane potential, ethanol, sodium selenite, heat shock, and an iron chelator. However, the timing is different, with the UPR^mt^ and MAPK^mt^ pathways being activated earlier. Other differences were apparent as well. *spg-7(RNAi)*, a classic activator of the UPR^mt^, also activates MAPK^mt^ but did not activate ESRE (and did not result in increased ROS level).

The genetic relationships between the pathways are also complex. For example, loss of UPR^mt^ function increased the activation of both the ESRE and the MAPK^mt^ pathways. One possible explanation is that the UPR^mt^ may respond more quickly or to lower levels of stress than the other pathways. Alternatively, the other pathways may serve to mitigate damage that the UPR^mt^ does not handle or may even clean up the damage that occurs due to UPR^mt^ activation. The presence of the ESRE motif in *atfs-1/ATF5* somewhat complicates this interpretation and suggests that expression of the UPR^mt^ may increase under ESRE-activating conditions. Further investigation of these genetic relationships is currently underway.

Previously, we reported that the ESRE network, which is conserved at least from *C. elegans* to mammals [28], played an unknown role in mitochondrial surveillance [26]. Here we show that a broad variety of treatments that disrupt mitochondrial ETC function result in ESRE gene activation. In addition, ESRE was activated by *spg-7(RNAi)*, although in this case the UPR^mt^ must be compromised. Furthermore, basal expression of ESRE genes increases when both mitochondrial SODs are mutated, suggesting that the presence of superoxide in this compartment is generally mitigated and that the absence of their removal activates surveillance pathways. These results confirm that ESRE functions as a bona fide mitochondrial surveillance program.

Unfortunately, our best efforts were unable to provide an unambiguous answer as to whether the ROS responsible for ESRE activation is superoxide, hydrogen peroxide, or both. There are three reasons for this. First, although hydrogen peroxide is able to easily cross subcellular membranes, mitochondrially located superoxide has been shown to escape mitochondria using the voltage-dependant anion channel [66]. Consequently, subcellular localization is insufficient to distinguish between the two. Second, the most commonly used fluorescence agent to detect superoxide, DHE, can be oxidized by either, despite yielding different products [51]. Although there is evidence to suggest that it is more effectively oxidized by superoxide [56], it appears that both can contribute to DHE fluorescence. Since there are currently no effective ways to deconvolve these fluorescent signals, a final answer to these questions may have to wait until better tools are available.

With these caveats in mind, the evidence available suggests to us that it is most likely to be superoxide. First, all of our results are consistent with this interpretation. In each case, activation of the ESRE network occurs after treatment that would generate superoxide or superoxide and H_2_O_2_, but only if mitochondrial damage occurs. Second, conditions that are expected to exacerbate superoxide production specifically, such as the combination of rotenone and N-acetylcysteine, strongly upregulate ESRE. Third, the correlation between measurement of activation of the *3XESRE*::GFP reporter and DHE staining across a gradient of exogenous H_2_O_2_ is much better than the correlation between either *3XESRE*::GFP or DHE with Amplex Red. Finally, the ability of a panel of known ESRE activators was tested to generate H_2_O_2_ showed generally low production. Taken as a whole, our data strongly suggest that ESRE activation is specifically due to the presence and amount of ROS, particularly superoxide.

Despite this, we think that it is unlikely that the ESRE network detects superoxide directly, since this molecule is short-lived, is very reactive, and is rapidly dismuted into hydrogen peroxide. Instead, it is more likely that the ESRE network responds to superoxide-mediated oxidation of some intracellular substance. This may explain how a short-lived molecule can trigger a pathway that requires a prolonged exposure (~ 2–6 h, depending on treatment) before endogenous gene expression can be consistently detected.

O’Rourke and colleagues have developed an interesting hypothesis that mitochondria have evolved to function in an optimized redox environment and that too great of a shift from this redox balance, in either direction, leads to increased ROS production [74, 75]. As might be expected, reductive stress can cause severe disruptions to cellular health, including loss of mitochondrial homeostasis, protein misfolding in the ER and the mitochondria due to inappropriate or absent disulfide bond formation, and ultimately proteostatic collapse [76–79]. At the tissue and organ level, damage can be profound or even lethal [41]. It is unsurprising that the organism would have a mechanism for handling reductive stress. Although it remains to be proven whether the ESRE network is that system, it is an intriguing possibility.

## Conclusions

It is increasingly clear that living organisms surveil their cellular functions to ensure the maintenance of homeostasis. In this article, we studied the activation of the ESRE network and its relationship with other mitochondrial surveillance pathways. Our research showed that perturbation of mitochondrial ETC results in ESRE gene activation due to increases in ROS. This response is also conserved in humans. While ROS are almost always linked to oxidative stress, we found that the ESRE pathway may serve as a readout for reductive stress instead. Enhanced activation of ESRE expression was observed in the absence of the UPR^mt^ and MAPK^mt^ pathways, which also surveil mitochondria. This relationship is made more complicated by the presence of an ESRE element in the promoter of *atfs-1* that is required for its expression. Since mitochondrial health is an important factor in many diseases, better understanding of the ESRE network is critical for understanding and improving numerous aspects of human health.

## Methods

### *C. elegans* strains

All *C. elegans* strains were maintained on nematode growth medium (NGM) seeded with *Escherichia coli* strain OP50 as a food source and were maintained at 20 °C [80] unless otherwise noted. *C. elegans* strains used in this study included N2 Bristol (wild type), WY703 (*fdIs2* {*3XESRE*::GFP; *pFF4*[*rol-6*(*su1006*)]}] [81], SJ4100 {*zcIs13* [*Phsp-6::*GFP]}, SLR115 {*dvIs67* [*Ptbb-6::*GFP + *Pmyo-3::*dsRed]}, NVK93 (*houIs002* {*pJY323*[*Phsp-16.1*::GFP]; *pRF4* [*rol-6*(*gf*)]}) [81], WY756 (*fdEx139* {*pJY312* [*Phsp-16.1(dd)*::GFP]; *pRF4*}) [81], GR2183 {*mgIs72* [*Prpt-3::*GFP + *dpy-5(+)*]}, SJ4005 {*zcIs4* [*Phsp-4::*GFP]}, CL2166 {*dvIs19* [*Pgst-4*::GFP::NLS]}, CF1553 {*muIs84* [*Psod-3*::GFP + *rol-6*(*su1006*)]}, ALF86 (*Pmyo-3::*Peredox*::unc-119*), NVK90 (*pink-1(tm1779)*; *houIs001* {*byEx655* [*Ppink-1*::PINK-1::GFP + *Pmyo-2*::mCherry]}), *atfs-1(cmh15),* GA186 [*sod-3(tm760)*], GA187 [*sod-1(tm776)*], GA416 [*sod-4(gk101)*], GA480 [*sod-2(gk257); sod-3(tm760)*], GA503 [*sod-5(tm1146)*], and RB1072 [*sod-2(ok1030)*], PHX1151 (*Patfs-1ΔESRE*::ATFS-1^WT^), NVK233 (*dvIs67; Patfs-1ΔESRE*::ATFS-1^WT^), NVK236 (*zcIs13; Patfs-1ΔESRE*::ATFS-1^WT^, line 1), and NVK237 (*zcIs13; Patfs-1ΔESRE*::ATFS-1^WT^, line 2).

Media conditions include NGM [80], a standard nematode growth medium. Prior to experiments, worms were synchronized by hypochlorite isolation of eggs from gravid adults, followed by hatching of eggs in S Basal. Six thousand synchronized L1 larvae were transferred onto 10-cm NGM plates seeded with *E. coli* OP50 or NGM plates supplemented with 25 μg/ml carbenicillin and 1 mM IPTG that were seeded with appropriate RNAi strains. After transfer, worms were grown at 20 °C for 50 h prior to use. Young adult worms were used for all assays unless otherwise noted.

### Bacterial strains

RNAi experiments in this study were done using RNAi-competent HT115 obtained from the Ahringer or Vidal RNAi library [82, 83]. All plasmids were sequence-verified prior to use.

### *C. elegans* chemical exposure assays

Prior to exposure to mitochondrial-damaging agents and/or antioxidants, synchronized young adult worms were washed from NGM plates seeded with OP50, and then resuspended in S Basal supplemented with one of the following: 50 μM antimycin A (Sigma), 50 μM rotenone (Sigma), 3 mM TTFA (Sigma), 10 mM sodium azide (Sigma), 20 μM CCCP (Sigma), 10% ethanol (Fisher), 1 mM phenanthroline (Sigma), 7 mM sodium selenite (Alfa Aesar), 1 mM H_2_O_2_ (Sigma), 60 μM tunicamycin (Thermo Fisher), 12.5 μM bortezomib (Thermo Fisher), 50 μM juglone (Sigma), 5 or 2.5 mM NAC (Acros Organics), 25 mM ascorbate (TCI), 5 mM TEMPOL (Cayman Chemical), and 10 μM mitoquinol (Cayman Chemical) in the presence of OP50. When specified, treatments were combined (e.g., rotenone/NAC, rotenone/NAC/ascorbate, etc.). Worms were imaged every 2 h for 20 h. Three biological replicates per experiment were performed, with ~ 400 worms per replicate.

### Quantitative reverse transcriptase PCR (qRT-PCR)

Six thousand young adult worms were used for RNA purification and subsequent qRT-PCR (performed as previously described [84]). Prior to RNA extraction, worms were grown on RNAi bacteria from L1 stage to young adult stage. Primer sequences are available upon request. For each experiment, at least 3 biological replicates were used.

### Imaging and fluorescence quantification

For visualization of the ALF86, CF1553, CL2166, GR2183, NVK90, NVK93, SJ4005, SJ4100, SLR115, WY703, and WY756 young adults exposed to mitochondrial-damaging agents in 96-well plates, Cytation 5 Cell Imaging Multi-Mode Reader (BioTek Instruments) was used. All the imaging was performed with identical settings. GFP quantifications were performed by using Gen5 3.0 software and via flow vermimetry on COPAS FP (Union Biometrica). Approximately four hundred worms were used for Cytation 5 imaging and ~ 1000 worms were used for flow vermimetry per biological replicate, and three biological replicates were used for each experiment.

### ROS quantifications

Superoxide was measured by using dihydroethidium (DHE) (Thermo Fisher) dye. Amplex Red assay was performed to measure hydrogen peroxide levels as described (Thermo Fisher). Worms were treated with mitochondrial-damaging agents for 10 h in 96-well plate before fluorescence measurement was taken. DHE was added at a final concentration of 3 μM and was let stained for 1 h in the dark. Worms were washed three times before reading to remove any remaining drugs or dye. Fluorescence intensities were recorded by using COPAS Biosort for DHE staining or Cytation 5 Cell Imaging Multi-Mode Reader (BioTek Instruments) at 545/590 nm for Amplex Red. For each experiment, at least 3 biological replicates were used with ~ 400 worms per replicate.

### Cell culture

RWPE-1 cells were maintained in defined keratinocyte SF media (Sigma), supplemented with human recombinant epidermal growth factor (rEGF) and bovine pituitary extract (BPE) (Gibco, Thermo Fisher). 16HBE cells were maintained in minimum essential medium eagle (MEM) supplemented with 10% HyClone fetal bovine serum (FBS) (GE Healthcare) and 1% nonessential amino acids (NEAA) (Gibco, Thermo Fisher). All media were supplemented with penicillin-streptomycin-glutamine (Gibco, Thermo Fisher) at a final concentration of 1%. All cells were maintained at 37 °C in a humidified 5% CO_2_ atmosphere. Non-supplemented MEM with 1% penicillin-streptomycin-glutamine was used for experiments. Cells were exposed to 20 μM CCCP or DMSO, alone or in combination with 0.5 mM TEMPOL (Cayman Chemical) or 2.5 μM MitoTEMPO (Cayman Chemical) in MEM with 1% penicillin-streptomycin for 16 h prior to RNA extraction and purification. For each experiment, at least 3 biological replicates of ~ 10^6^ cells were used.

### Statistical analysis

RStudio (version 3.6.3) was used to perform statistical analysis. One-way analysis of variance (ANOVA) was performed to calculate the significance of a treatment when there were three or more groups in the experimental setting. To follow, Dunnett’s test (R package DescTools, version 0.99.34) was performed to calculate statistical significance or *p* values between each group of the statistically significant experimental results. Student’s *t* test analysis was performed to calculate the *p* values when comparing two groups in an experimental setting. Both Dunnett’s test and Student’s *t* test results were indicated in graphs, NS not significant, *p < 0.05, **p < 0.01, and ***p < 0.001.

Figure 3c and Fig. S10E (Additional File 1) are generated with R package ggplot2 (version 3.3.0). Correlation coefficients and *p* values between DHE or Amplex Red fluorescence and reporters’ expressions were calculated and indicated on graphs. Z-statistics were calculated to infer significance between two means of the linear models for each interaction.

## Supplementary information

**Additional file 1: Fig. S1**. Expression of ESRE endogenous genes were increased upon treatment with rotenone. Expression of a panel of ESRE genes in wild type N2 worms were measured at 2 h, 4 h, and 8 h upon treatment with 50 μM rotenone. **Fig. S2.***spg-7(RNAi)* did not activate the ESRE reporters. (A) Quantification of GFP fluorescence for *3XESRE*::GFP, *Phsp-6*::GFP, *Ptbb-6*::GFP, and *Phsp-16.1*::GFP reporters after being reared for two days on empty vector (*EV*) or *spg-7(RNAi)*-expressing *E.coli*. (B) Fluorescent images of *C. elegans* carrying the ESRE native reporter (*Phsp-16.1*::GFP) after 10 h treatment with a panel of mitochondria-damaging agents. Three biological replicates with ~ 400 worms/replicate were analyzed. Error bars represent SEM. *p-*values were determined from Student’s *t*-test. All fold changes were normalized to *EV* control. NS: not significant, *** *p* < 0.001. **Fig. S3.** UPR^mt^ and MAPK^mt^, but not ESRE, pathways were activated by RNAi targeting mitochondrial components. Quantification of GFP fluorescence for (A) *3XESRE*::GFP, (B) *Phsp-6*::GFP, or (C) *Ptbb-6*::GFP reporters that were reared on *E. coli* containing empty vector (*EV*), *cco-1(RNAi)*, *mrps-5(RNAi)*, *tomm-20(RNAi)*, or *tomm-22(RNAi)*. Three biological replicates with ~ 400 worms/replicate were analyzed. Error bars represent SEM. *p-*values were determined from one-way ANOVA, followed by Dunnett’s test. All fold changes were normalized to *EV* control. NS: not significant, * *p* < 0.05, ** *p* < 0.01, *** *p* < 0.001. **Fig. S4.** Bortezomib and tunicamycin did not induce the ESRE reporter. (A, B) Fluorescent images of *3XESRE:*:GFP (top) or (A) *Prpt-3*::GFP (bottom) or (B) *Phsp-4*::GFP (bottom) after treatment with DMSO or (A) 12.5 μM bortezomib or (B) 60 μM tunicamycin for 10 h, correspondingly. Representative images are shown; three biological replicates with ~ 400 worms/replicate were analyzed. **Fig. S5.** The combination of rotenone and NAC did not affect UPR^mt^ and MAPK^mt^ expressions. Fluorescent images of *C. elegans* strains with (A) *Phsp-6*::GFP or (B) *Ptbb-6*::GFP reporters that were treated with vehicle (DMSO) (left) or 50 μM rotenone (right) with or without 25 mM ascorbate (Asc), 2.5 mM NAC, or ascorbate and NAC combination (NAC/Asc) for 8 h. Quantification of GFP fluorescence for (C) *Phsp-6*::GFP or (D) *Ptbb-6*::GFP reporters under the same conditions as in (A-B). Three biological replicates with ~ 400 worms/replicate were analyzed. Error bars represent SEM. *p-*values were determined from one-way ANOVA, followed by Dunnett’s test. GFP values were normalized to DMSO. NS: not significant, * *p* < 0.05, ** *p* < 0.01. **Fig. S6**. *spg-7(RNAi)* did not induce the production of superoxide. Quantification of ROS level (based on DHE fluorescence) of N2 worms reared on *E. coli* expressing *EV* or *spg-7(RNAi)*. Three biological replicates with ~ 400 worms/replicate were analyzed. Error bars represent SEM. *p-*values were determined from Student’s *t*-test. Superoxide level was normalized to *EV*. NS: not significant. **Fig. S7.** Juglone induced ESRE genes expression. (A) Quantification of DHE fluorescence of N2 worms treated with 50 μM juglone for 8 h, normalized to ethanol control. (B) Expression of ESRE genes in N2 worms treated with 50 μM juglone for 8 h. As positive controls, *gst-4* (SKN-1 target) and *cdr-4* (UPR^mt^) genes were used. Three biological replicates with ~ 1000 worms/replicate for fluorescence quantification and ~ 8000 worms/replicate for quantitative RT-PCR were analyzed. Error bars represent SEM. *p-*values were determined from Student’s *t*-test. NS: not significant, * *p* < 0.05, ** *p* < 0.01. **Fig. S8.** Mitochondrial-damaging agents failed to activate oxidative stress reporter *Pgst-4*::GFP. Quantification of GFP fluorescence for *Pgst-4*::GFP reporters after 10 h treatment with a panel of mitochondria-damaging agents or juglone as positive control. Three biological replicates with ~ 400 worms/replicate were analyzed. Error bars represent SEM. *p-*values were determined from one-way ANOVA, followed by Dunnett’s test. All fold changes were normalized to DMSO or ethanol control (for juglone). NS: not significant, *** *p* < 0.001. **Fig. S9.** Loss of mitochondrial superoxide dismutases enhanced basal ESRE expression. Basal expression of ESRE genes in *sod-1*, *sod-5*, *sod-2*, *sod-3*, *sod-2;sod-3*, and *sod-4* mutants, normalized to wild type N2 animals. Three biological replicates with ~ 8000 worms/replicate were analyzed. Error bars represent SEM. *p-*values were derived from one-way ANOVA, followed by Dunnett’s test. * *p* < 0.05, ** *p* < 0.01, *** *p* < 0.001. **Fig. S10.** Hydrogen peroxide level did not correlate with mitochondrial surveillance pathways’ activation. (A) Fluorescent images of *C. elegans* strains with *3XESRE*::GFP (left), *Phsp-6*::GFP (middle), or *Ptbb-6*::GFP (right) reporters following treatment with 1 mM H_2_O_2_. Quantification of (B) ROS (based on DHE fluorescence) or (C) hydrogen peroxide (using Amplex Red staining) levels after treatment with 1 mM H_2_O_2_. (D) Quantification of H_2_O_2_ content based on Amplex Red staining after exposure to a panel of mitochondrial-damaging molecules or vehicle (DMSO). (E) Correlation of hydrogen peroxide content (Amplex Red) with reporters’ expression. Representative images for (A) are shown; exposure length was 10 h. Three biological replicates with ~ 400 worms/replicate were analyzed. Error bars represent SEM. *p-*values were determined from (B-C) Student’s *t*-test and (D) one-way ANOVA, followed by Dunnett’s test. All fold changes were normalized to vehicle control. NS: not significant, * *p* < 0.05, ** *p* < 0.01, *** *p* < 0.001. **Fig. S11.** ESRE expression upon induction with hydrogen peroxide is well-correlated with DHE, but not Amplex Red. (A) Amplex Red fluorescence after treatment with a gradient of H_2_O_2_ concentrations. (B) DHE fluorescence following treatment with a gradient of H_2_O_2_ concentrations. (C) Correlation of *3XESRE*::GFP fluorescence with Amplex Red-measured H_2_O_2_ content. (D) Correlation of *3XESRE*::GFP fluorescence with DHE fluorescence upon treatment with H_2_O_2_. Exposure length was 8 h. Three biological replicates with ~ 1000 worms/replicate were analyzed. All fold changes were normalized to no H_2_O_2_ control. **Fig. S12.** Loss of UPR^mt^ and MAPK^mt^ regulators resulted in early activation of the ESRE pathway. (A) Quantification of GFP fluorescence for *3XESRE*::GFP reporter reared on *E. coli* expressing empty vector (*EV*), *atfs-1(RNAi)*, *pmk-3(RNAi)*, or *pmk-3(RNAi);atfs-1(RNAi)* after 6 h treatment with vehicle (DMSO) or 50 μM rotenone. GFP values were normalized to *EV* on DMSO. (B) Fluorescent images of *C. elegans* carrying the ESRE minimal reporter (*3XESRE*::GFP, left) or ESRE native reporter (*Phsp-16.1*::GFP, right) reared on *E. coli* expressing empty vector (*EV*), *atfs-1(RNAi)*, *spg-7(RNAi)*, or *spg-7(RNAi); atfs-1(RNAi)* for 2 days. Three biological replicates with ~ 400 worms/replicate were analyzed. Error bars represent SEM. *p-*values were determined from one-way ANOVA, followed by Dunnett’s test. NS: not significant, * *p* < 0.05, *** *p* < 0.001. **Fig. S13.** UPR^mt^ restricts MAPK^mt^ basal and induced expression. (A) Expression of MAPK^mt^ endogenous target genes in wild type N2 worms reared on *E. coli* expressing *atfs-1(RNAi)* or *spg-7(RNAi)*. (B) Expression of MAPK^mt^ endogenous target genes in wild type worms reared on *E. coli* expressing empty vector (*EV*), *atfs-1(RNAi)*, *pmk-3(RNAi)*, or *pmk-3(RNAi); atfs-1(RNAi)*. (C) Expression of MAPK^mt^ endogenous target genes in *Δatfs-1(cmh15)* null mutant reared on *E. coli.* Three biological replicates with ~ 6000 worms/replicate were analyzed. All fold changes were normalized to *EV*. Error bars represent SEM. *p-*values were derived from (A,B) one-way ANOVA, followed by Dunnett’s test or (C) *t*-test. NS: not significant, * *p* < 0.05, ** *p* < 0.01, *** *p* < 0.001. **Fig. S14.** ESRE motif in *atfs-1* is necessary for full *atfs-1* expression. (A) Expression of UPR^mt^ endogenous target genes in N2 wild type or *Patfs-1ΔESRE*::ATFS-1^WT^ worms on basal level. Fold changes were normalized to N2. (B) Fold changes of MAPK^mt^ endogenous target genes’ expressions in *Patfs-1ΔESRE*::ATFS-1^WT^ worms, compared to N2 worms. Both strains were reared on *E. coli* expressing *spg-7(RNAi)*. Three biological replicates with ~ 6000 worms/replicate were analyzed. Error bars represent SEM. *p-*values were derived from *t*-test. NS: not significant, * *p* < 0.05, ** *p* < 0.01.

**Additional file 2: Table S1.** Responses of mitochondrial surveillance pathway reporters to additional abiotic stressors. Summary of the activation of mitochondrial surveillance pathways after treatment with a panel of insults for 10 h, previously reported to activate ESRE. (−) indicates lack of response, plus (+), (++), or (+++) indicates weak, medium, or strong response, respectively. Three biological replicates with ~ 400 worms/replicate were analyzed. Responses were measured qualitatively.

## Data Availability

The datasets supporting the conclusions of this article are included within the article and its additional files.
