# Supplementary material for: The evolutionarily conserved ESRE stress response network is activated by ROS and mitochondrial damage
Source: BMC Biol. 2020 Jun 29;18:74. doi: 10.1186/s12915-020-00812-5 (PMC7322875; doi:10.1186/s12915-020-00812-5)
Supplement: Supplementary file 1 — Additional file 1: Fig. S1. Expression of ESRE endogenous genes were increased upon treatment with rotenone. Expression of a panel of ESRE genes in wild type N2 worms were measured at 2 h, 4 h, and 8 h upon treatment with 50 μM rotenone. Fig. S2.spg-7(RNAi) did not activate the ESRE reporters. (A) Quantification of GFP fluorescence for 3XESRE::GFP, Phsp-6::GFP, Ptbb-6::GFP, and Phsp-16.1::GFP reporters after being reared for two days on empty vector (EV) or spg-7(RNAi)-expressing E.coli. (B) Fluorescent images of C. elegans carrying the ESRE native reporter (Phsp-16.1::GFP) after 10 h treatment with a panel of mitochondria-damaging agents. Three biological replicates with ~ 400 worms/replicate were analyzed. Error bars represent SEM. p-values were determined from Student’s t-test. All fold changes were normalized to EV control. NS: not significant, *** p < 0.001. Fig. S3. UPRmt and MAPKmt, but not ESRE, pathways were activated by RNAi targeting mitochondrial components. Quantification of GFP fluorescence for (A) 3XESRE::GFP, (B) Phsp-6::GFP, or (C) Ptbb-6::GFP reporters that were reared on E. coli containing empty vector (EV), cco-1(RNAi), mrps-5(RNAi), tomm-20(RNAi), or tomm-22(RNAi). Three biological replicates with ~ 400 worms/replicate were analyzed. Error bars represent SEM. p-values were determined from one-way ANOVA, followed by Dunnett’s test. All fold changes were normalized to EV control. NS: not significant, * p < 0.05, ** p < 0.01, *** p < 0.001. Fig. S4. Bortezomib and tunicamycin did not induce the ESRE reporter. (A, B) Fluorescent images of 3XESRE::GFP (top) or (A) Prpt-3::GFP (bottom) or (B) Phsp-4::GFP (bottom) after treatment with DMSO or (A) 12.5 μM bortezomib or (B) 60 μM tunicamycin for 10 h, correspondingly. Representative images are shown; three biological replicates with ~ 400 worms/replicate were analyzed. Fig. S5. The combination of rotenone and NAC did not affect UPRmt and MAPKmt expressions. Fluorescent images of C. elegans strains with ( [file 12915_2020_812_MOESM1_ESM.pdf]

Additional File 1 for:

The Evolutionarily Conserved ESRE Stress Response Network is Activated by ROS and Mitochondrial Damage

Tjahjono, E., McAnena A. & Kirienko, N.V.*

* To whom correspondence should be addressed.
Tel: (713) 348-2581
Email: kirienko@rice.edu

This PDF includes:

Figures S1-S14

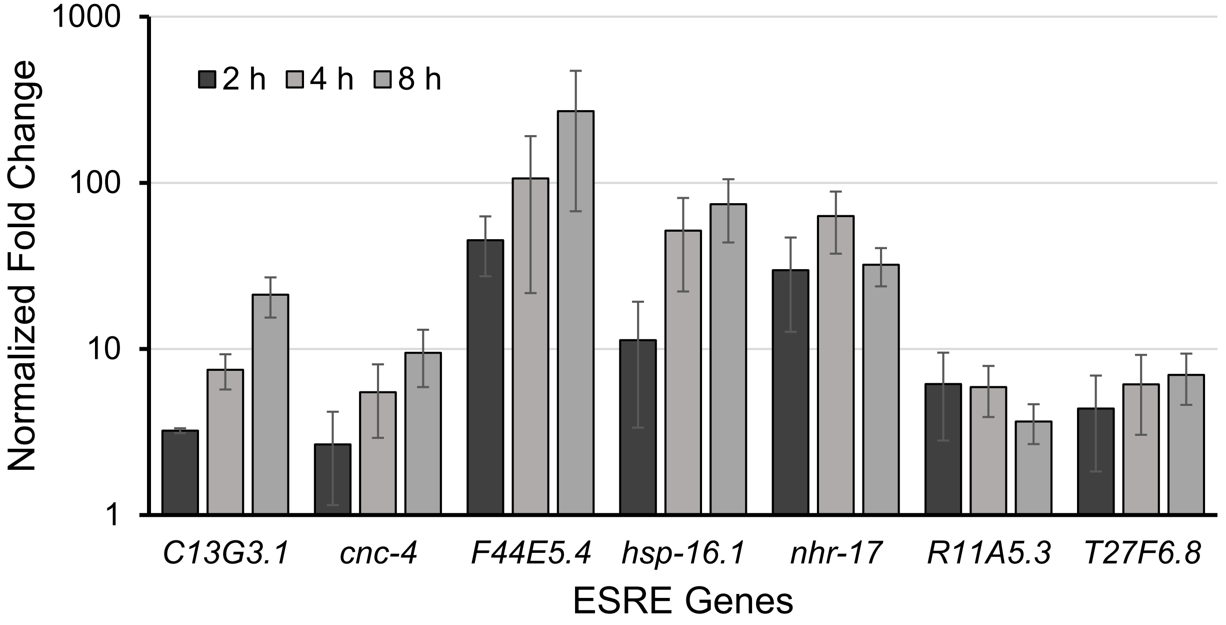

1000
100
10
1
Normalized Fold Change
2 h
4 h
8 h
C13G3.1
cnc-4
F44E5.4
hsp-16.1
nhr-17
R11A5.3
T27F6.8
ESRE Genes

Figure S1

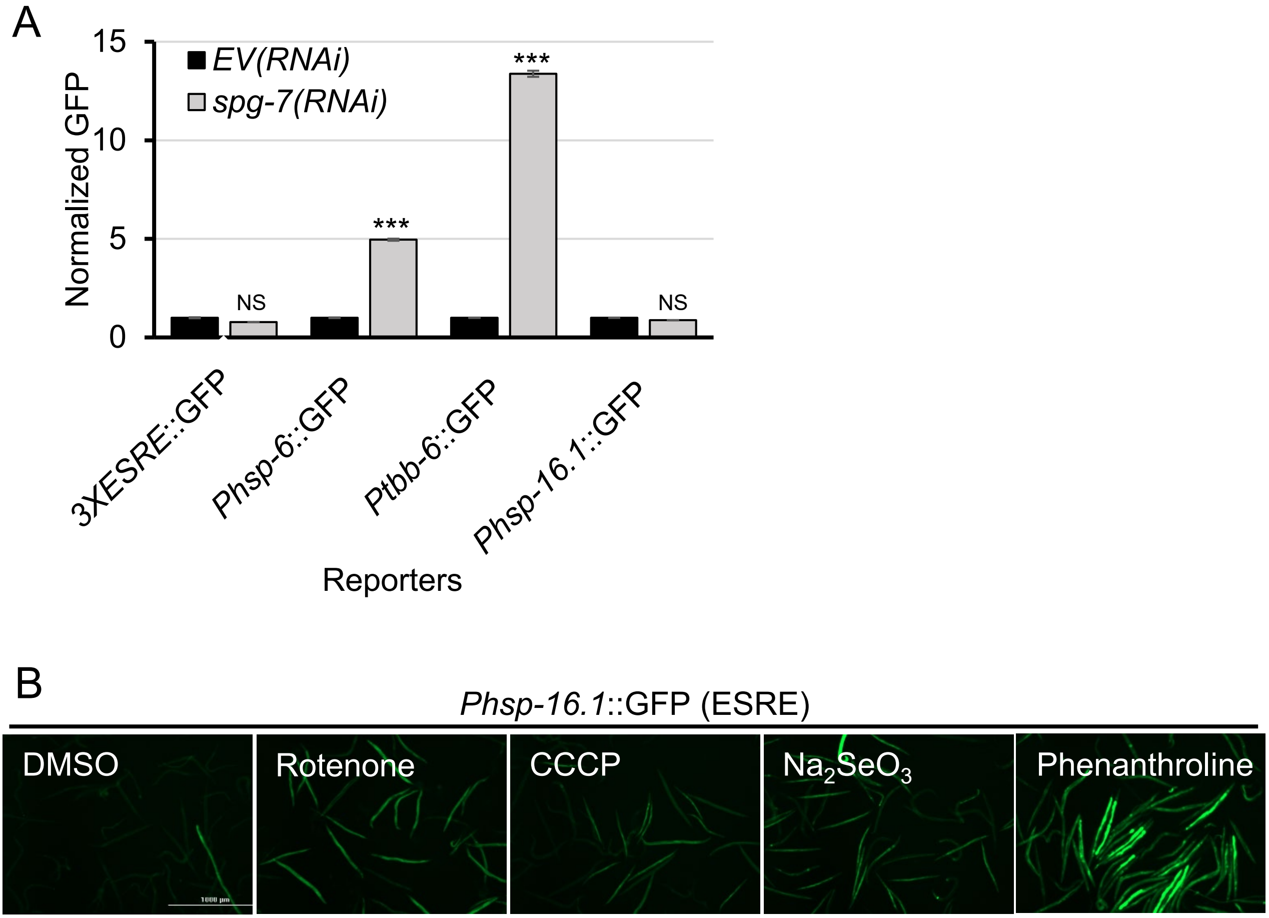
A
15
10
5
0
Normalized GFP
EV(RNAi)
spg-7(RNAi)
***
***
NS
NS
3XESRE::GFP
Phsp-6::GFP
Ptbb-6::GFP
Phsp-16.1::GFP
Reporters
B
Phsp-16.1::GFP (ESRE)
DMSO
Rotenone
CCCP
Na2SeO3
Phenanthroline

Figure S2

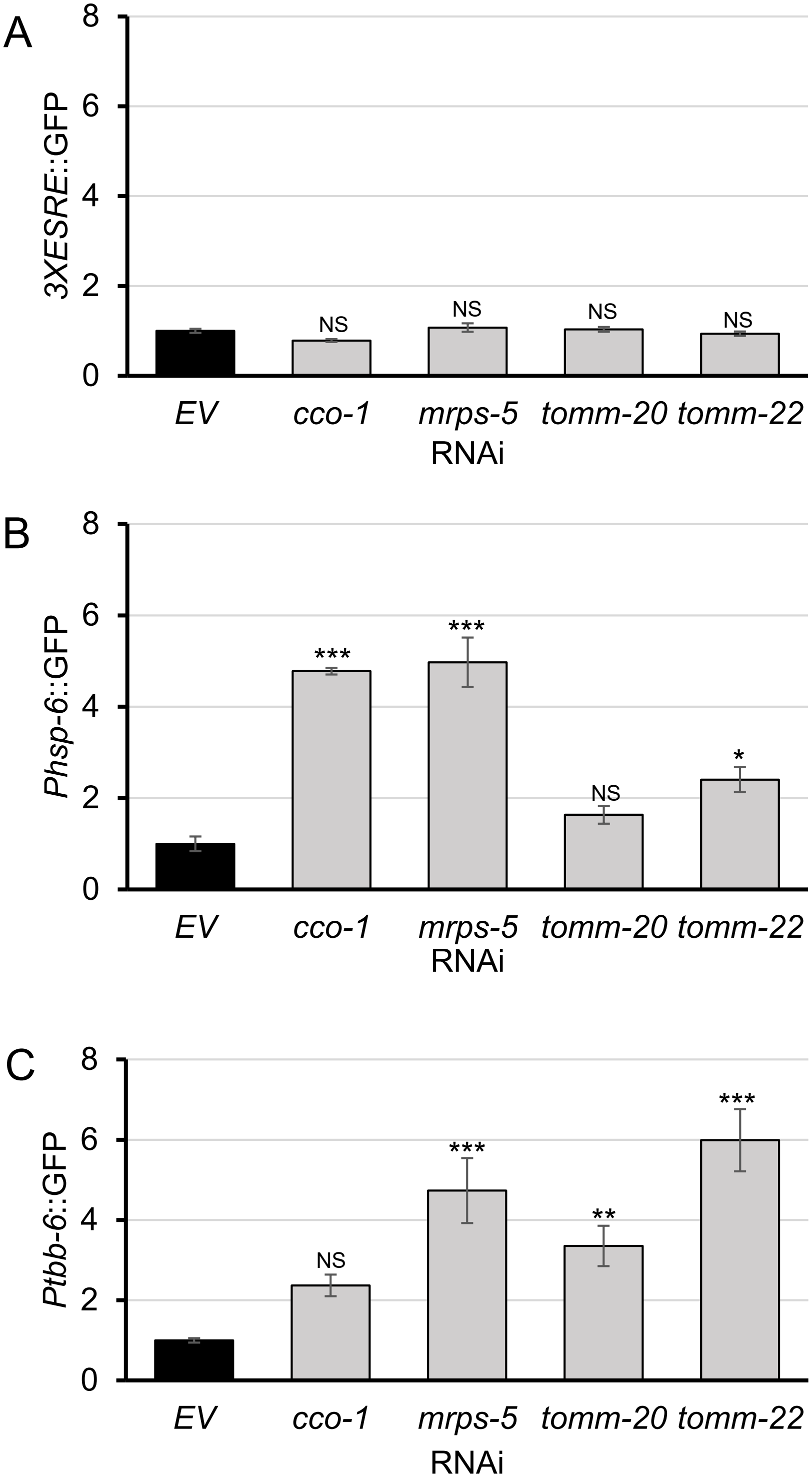
A
8
6
4
2
0
3XESRE::GFP
NS
NS
NS
NS
EV
cco-1
mrps-5
tomm-20
tomm-22
RNAi
B
8
6
4
2
0
Phsp-6::GFP
***
***
NS
*
EV
cco-1
mrps-5
tomm-20
tomm-22
RNAi
C
8
6
4
2
0
Ptbb-6::GFP
NS
***
**
***
EV
cco-1
mrps-5
tomm-20
tomm-22
RNAi

Figure S3

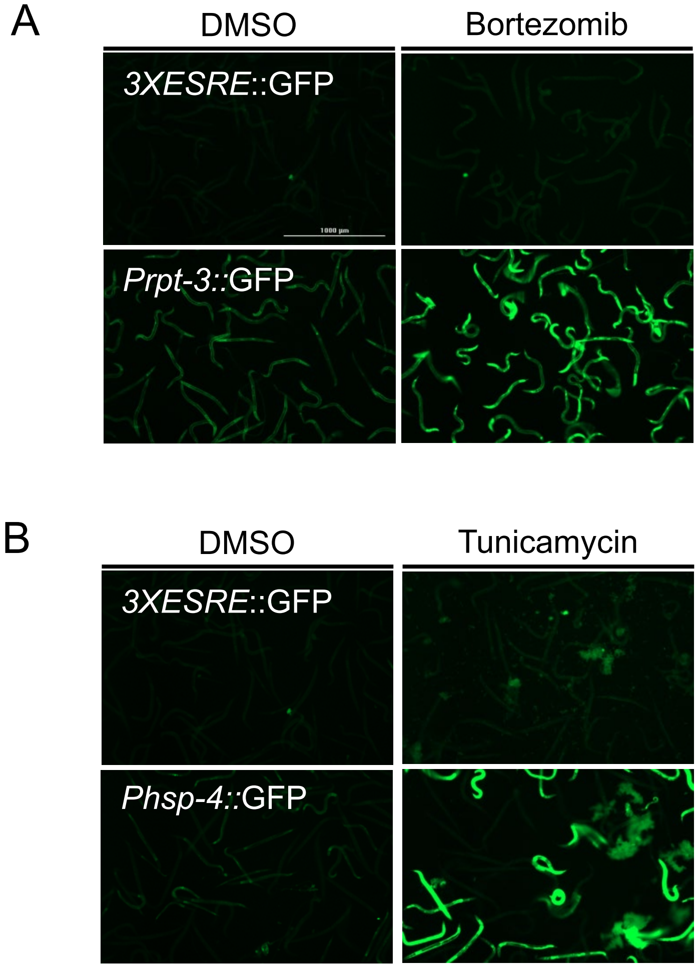
A
DMSO
Bortezomib
3XESRE::GFP
Prpt-3::GFP
B
DMSO
Tunicamycin
3XESRE::GFP
Phsp-4::GFP

Figure S4

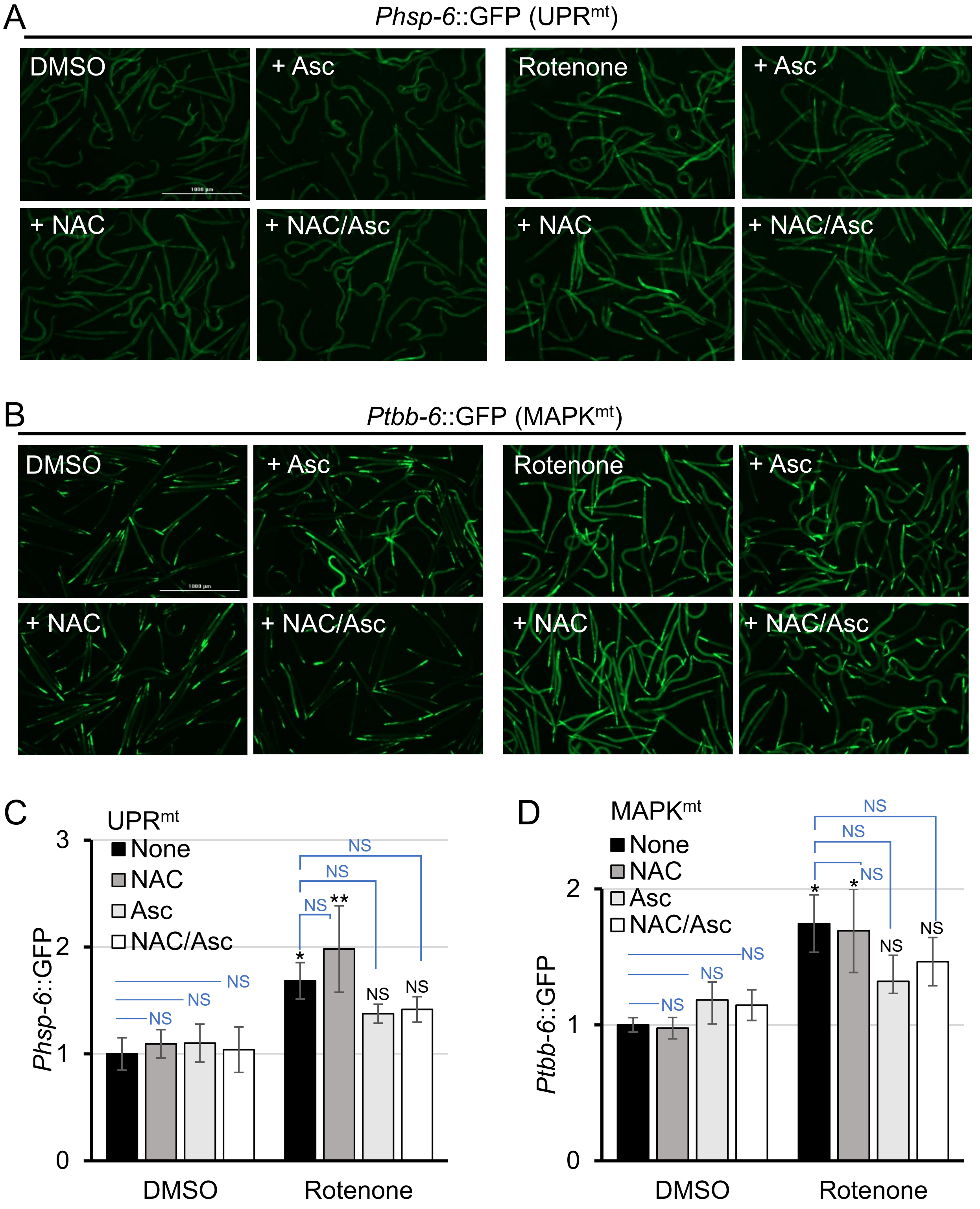
A
Phsp-6::GFP (UPRmt)
DMSO
+ Asc
Rotenone
+ Asc
+ NAC
+ NAC/Asc
+ NAC
+ NAC/Asc
B
Ptbb-6::GFP (MAPKmt)
DMSO
+ Asc
Rotenone
+ Asc
+ NAC
+ NAC/Asc
+ NAC
+ NAC/Asc
C
UPRmt
None
NAC
Asc
NAC/Asc
Phsp-6::GFP
DMSO
Rotenone
NS
D
MAPKmt
None
NAC
Asc
NAC/Asc
Ptbb-6::GFP
DMSO
Rotenone
NS

Figure S5

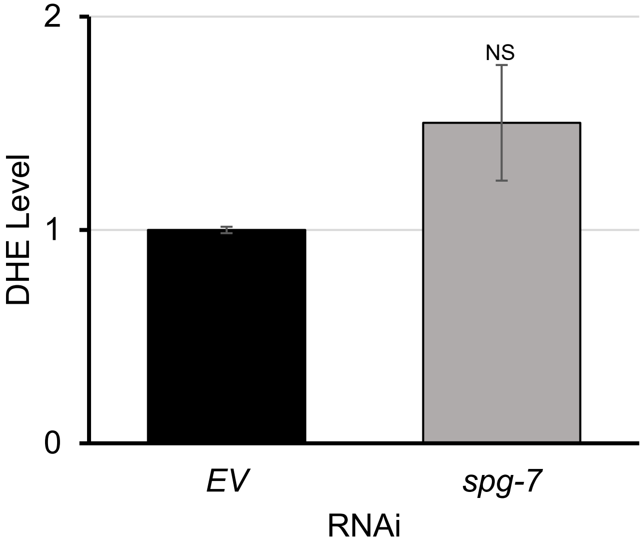

Figure S6

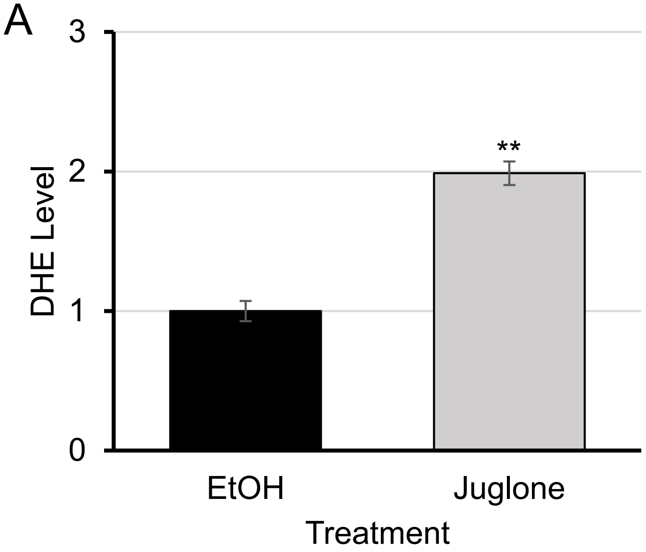
A
3
2
1
0
DHE Level
**
EtOH
Juglone
Treatment

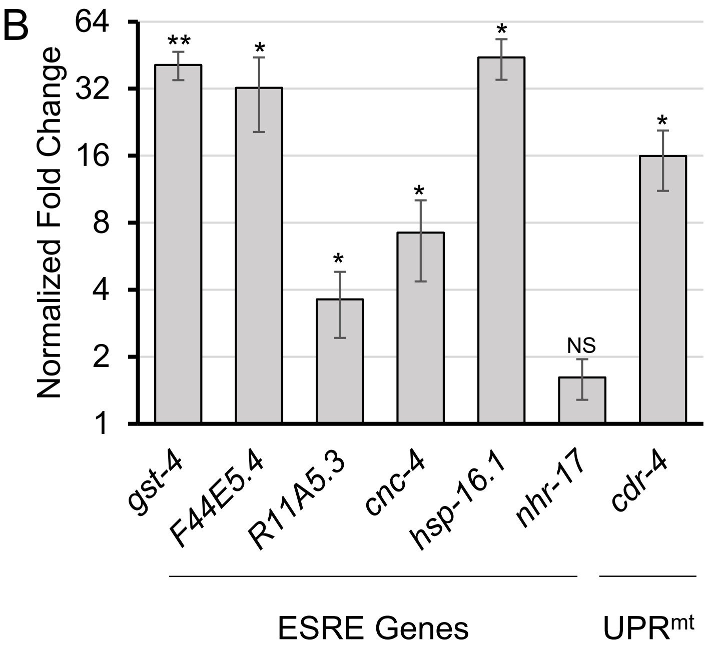
B
64
32
16
8
4
2
1
Normalized Fold Change
**
*
*
*
*
NS
*
gst-4
F44E5.4
R11A5.3
cnc-4
hsp-16.1
nhr-17
cdr-4
ESRE Genes
UPRmt

Figure S7

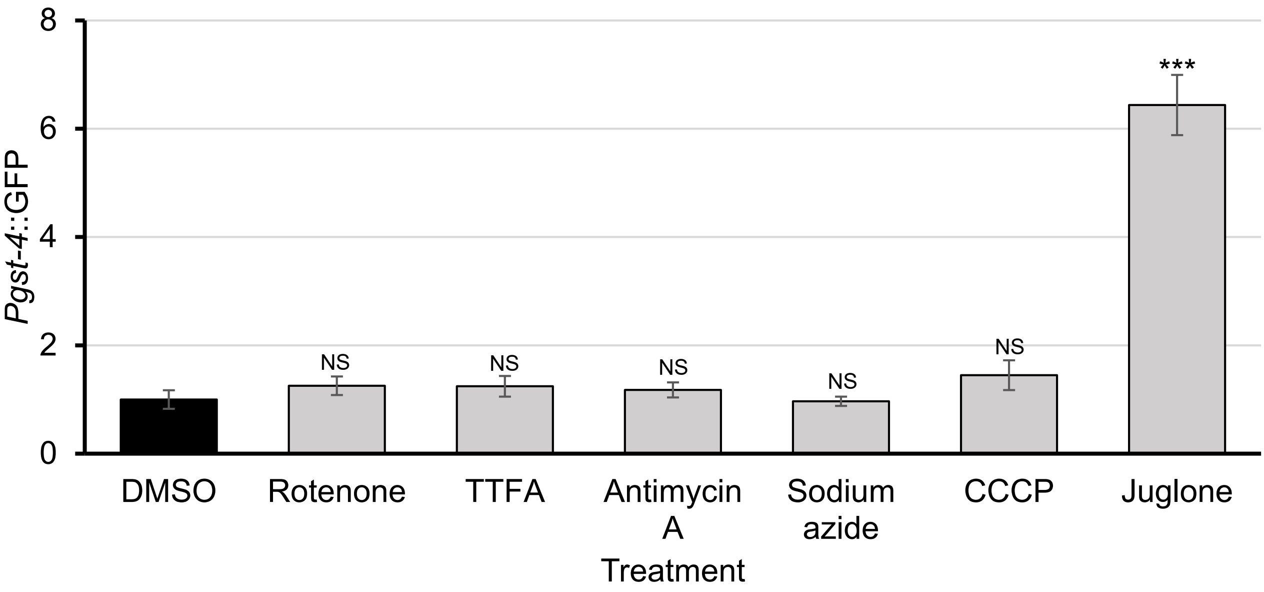

8
6
4
2
0
Pgst-4::GFP
***
NS
NS
NS
NS
NS
DMSO
Rotenone
TTFA
Antimycin A
Sodium azide
CCCP
Juglone
Treatment

Figure S8

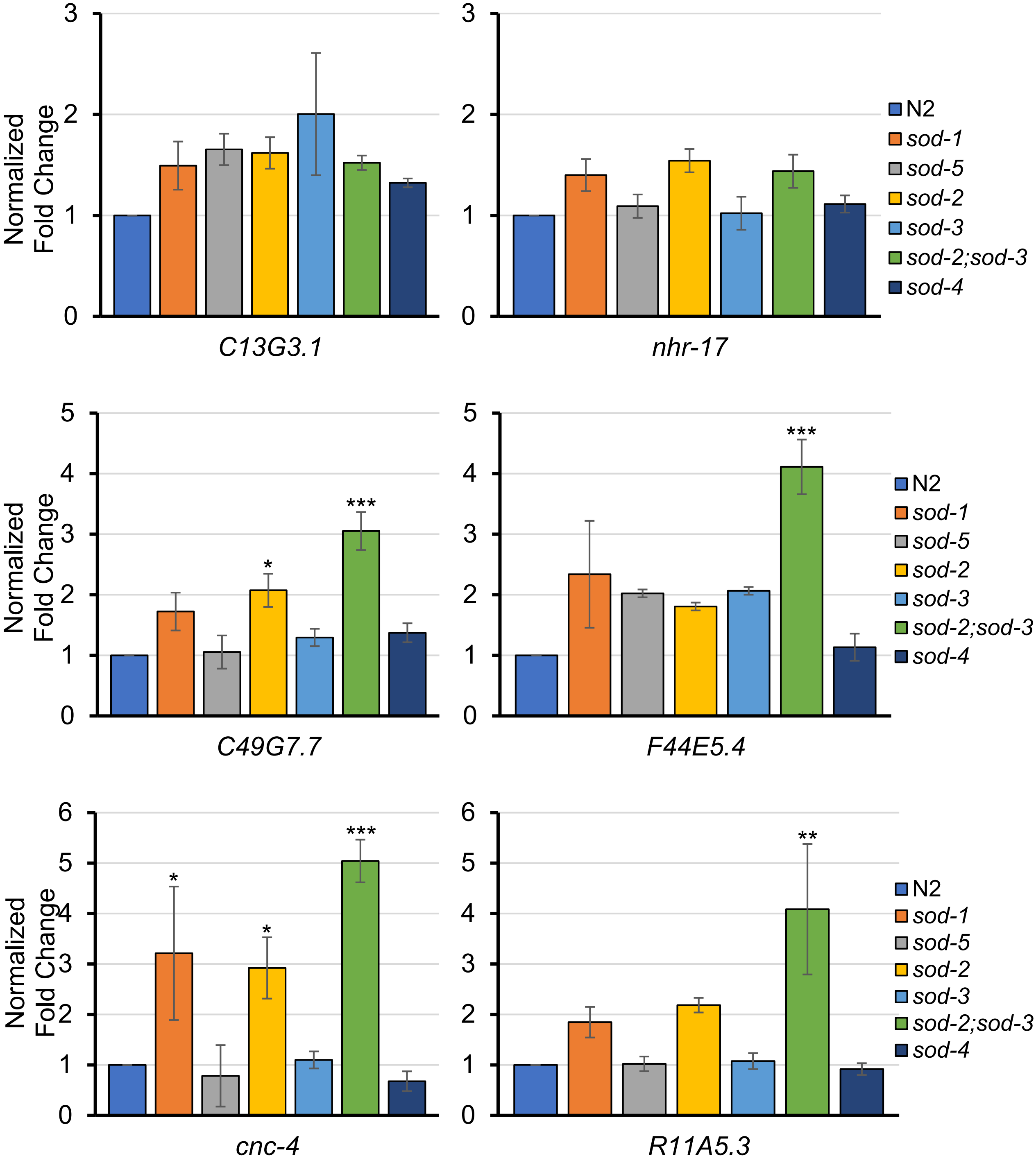
Normalized Fold Change
C13G3.1
nhr-17
C49G7.7
F44E5.4
cnc-4
R11A5.3
N2
sod-1
sod-5
sod-2
sod-3
sod-2;sod-3
sod-4
*
**
***

Figure S9

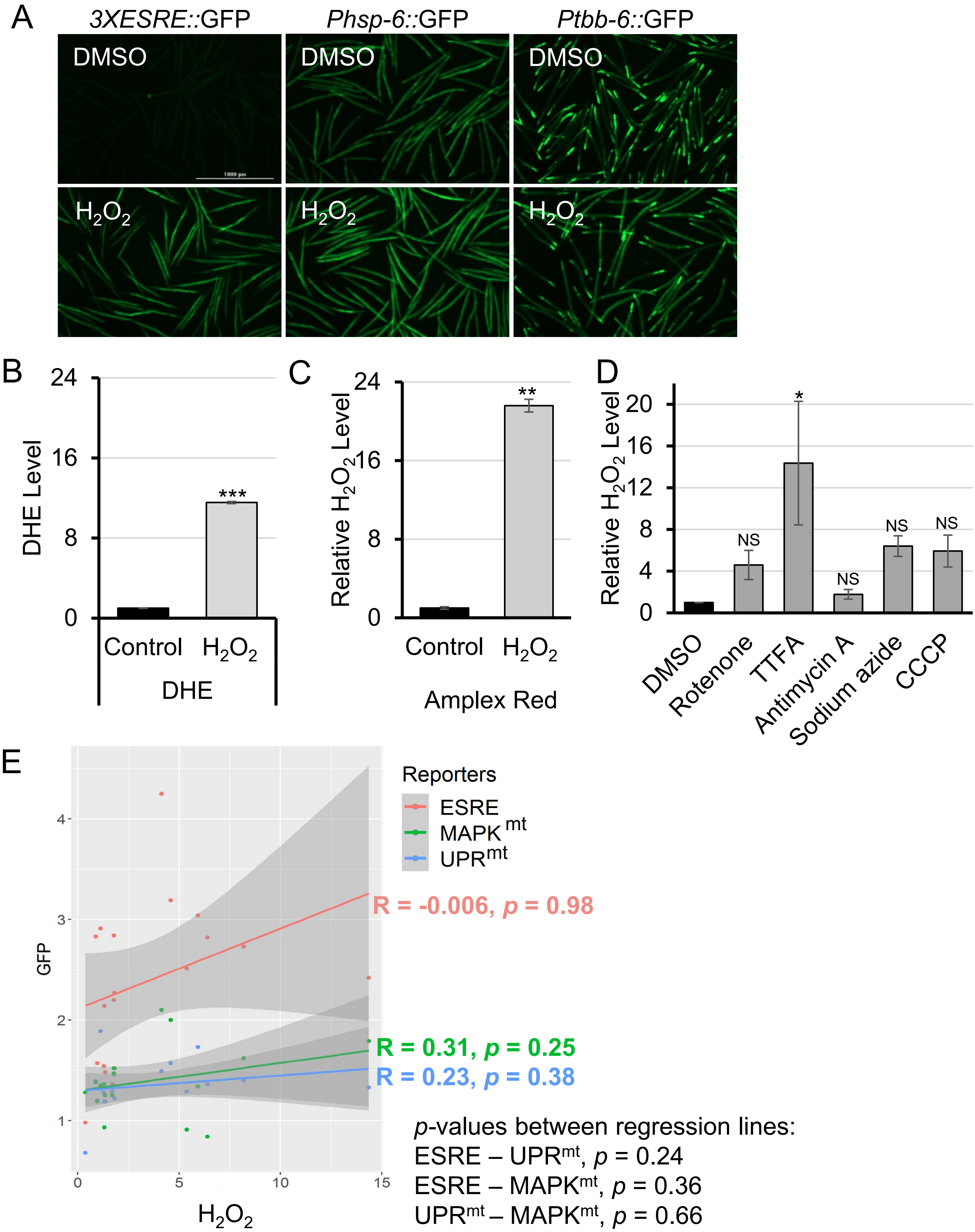

A
3XESRE::GFP
Phsp-6::GFP
Ptbb-6::GFP
DMSO
H2O2
B
DHE Level
24
16
8
0
***
Control
H2O2
DHE
C
Relative H2O2 Level
**
Amplex Red
D
20
12
4
*
NS
DMSO
Rotenone
TTFA
Antimycin A
Sodium azide
CCCP
E
GFP
Reporters
ESRE
MAPK mt
UPRmt
R = -0.006, p = 0.98
R = 0.31, p = 0.25
R = 0.23, p = 0.38
p-values between regression lines:
ESRE – UPRmt, p = 0.24
ESRE – MAPKmt, p = 0.36
UPRmt – MAPKmt, p = 0.66

Figure S10

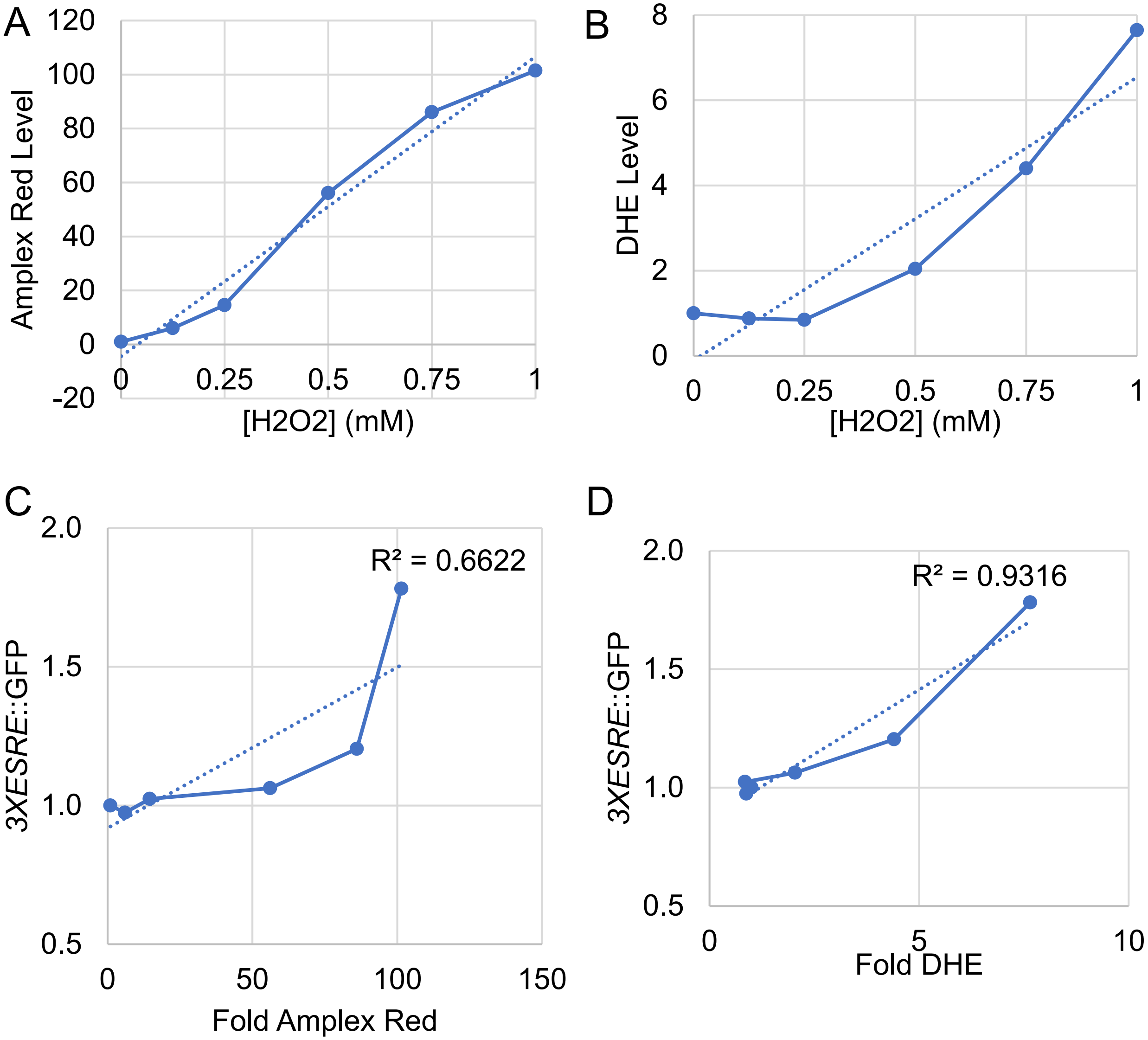

A
Amplex Red Level
120
100
80
60
40
20
0
-20
0
0.25
0.5
0.75
1
[H2O2] (mM)
B
DHE Level
8
6
4
2
0
0
0.25
0.5
0.75
1
[H2O2] (mM)
C
3XESRE::GFP
2.0
1.5
1.0
0.5
R² = 0.6622
0
50
100
150
Fold Amplex Red
D
3XESRE::GFP
2.0
1.5
1.0
0.5
R² = 0.9316
0
5
10
Fold DHE

Figure S11

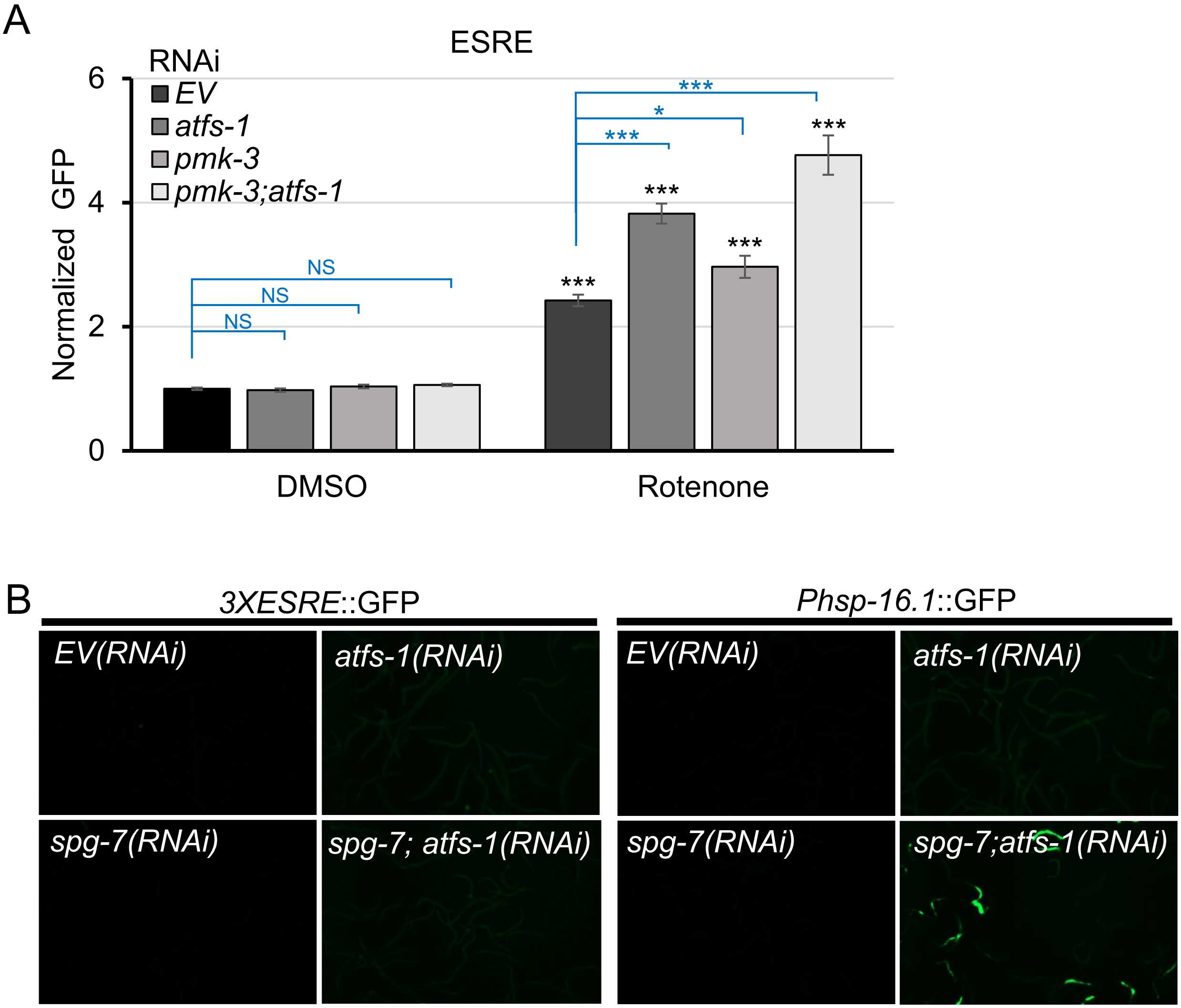

A
ESRE
RNAi
EV
atfs-1
pmk-3
pmk-3;atfs-1
Normalized GFP
6
4
2
0
NS
NS
NS
***
*
***
***
***
***
***
DMSO
Rotenone
B
3XESRE::GFP
Phsp-16.1::GFP
EV(RNAi)
atfs-1(RNAi)
spg-7(RNAi)
spg-7; atfs-1(RNAi)
EV(RNAi)
atfs-1(RNAi)
spg-7(RNAi)
spg-7;atfs-1(RNAi)

Figure S12

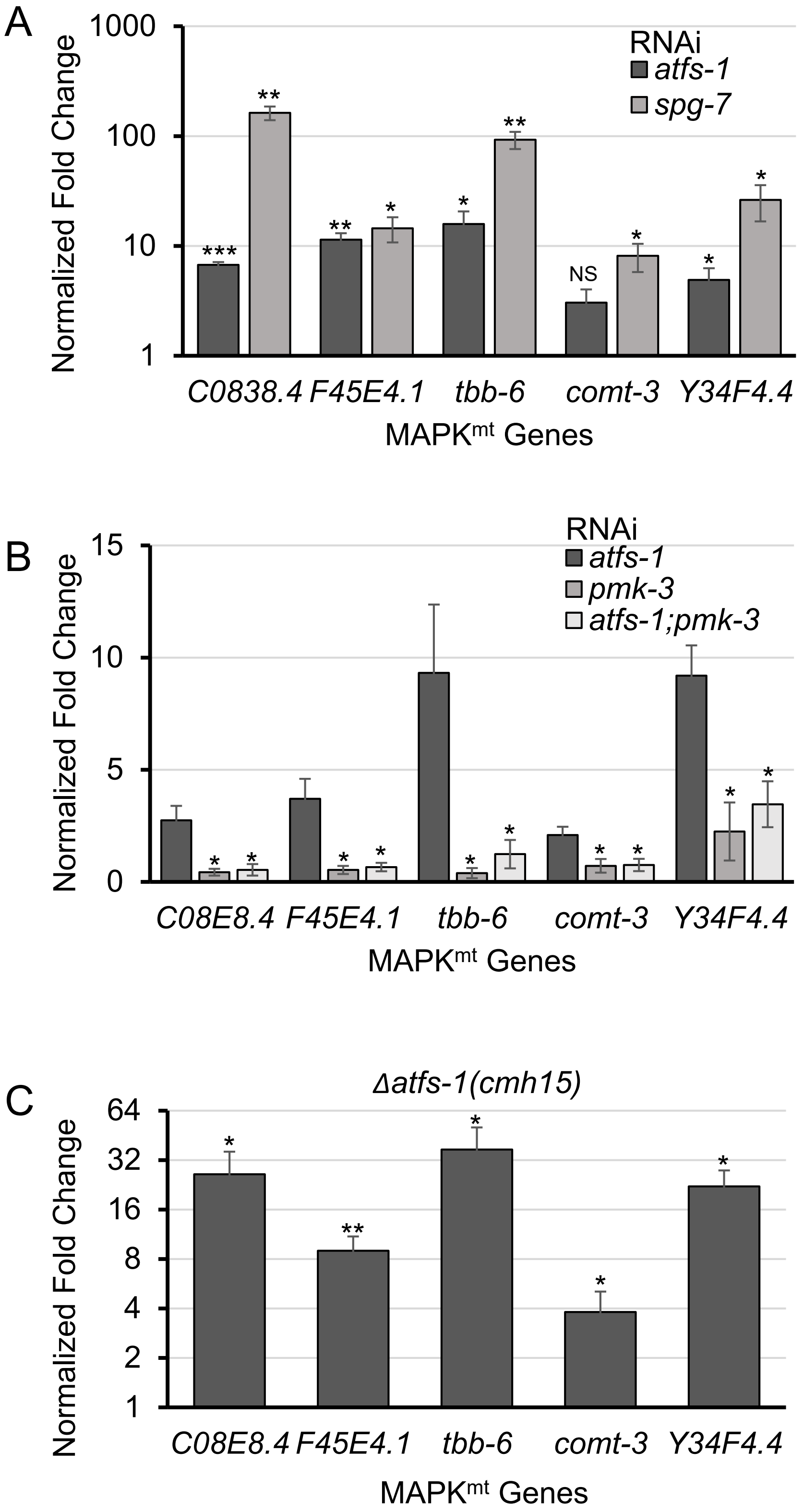
A
Normalized Fold Change
1000
100
10
1
RNAi
atfs-1
spg-7
***
**
**
*
*
**
NS
*
*
*
C0838.4
F45E4.1
tbb-6
comt-3
Y34F4.4
MAPKmt Genes
B
Normalized Fold Change
15
10
5
0
RNAi
atfs-1
pmk-3
atfs-1;pmk-3
C08E8.4
F45E4.1
tbb-6
comt-3
Y34F4.4
MAPKmt Genes
C
Δatfs-1(cmh15)
Normalized Fold Change
64
32
16
8
4
2
1
C08E8.4
F45E4.1
tbb-6
comt-3
Y34F4.4
MAPKmt Genes

Figure S13

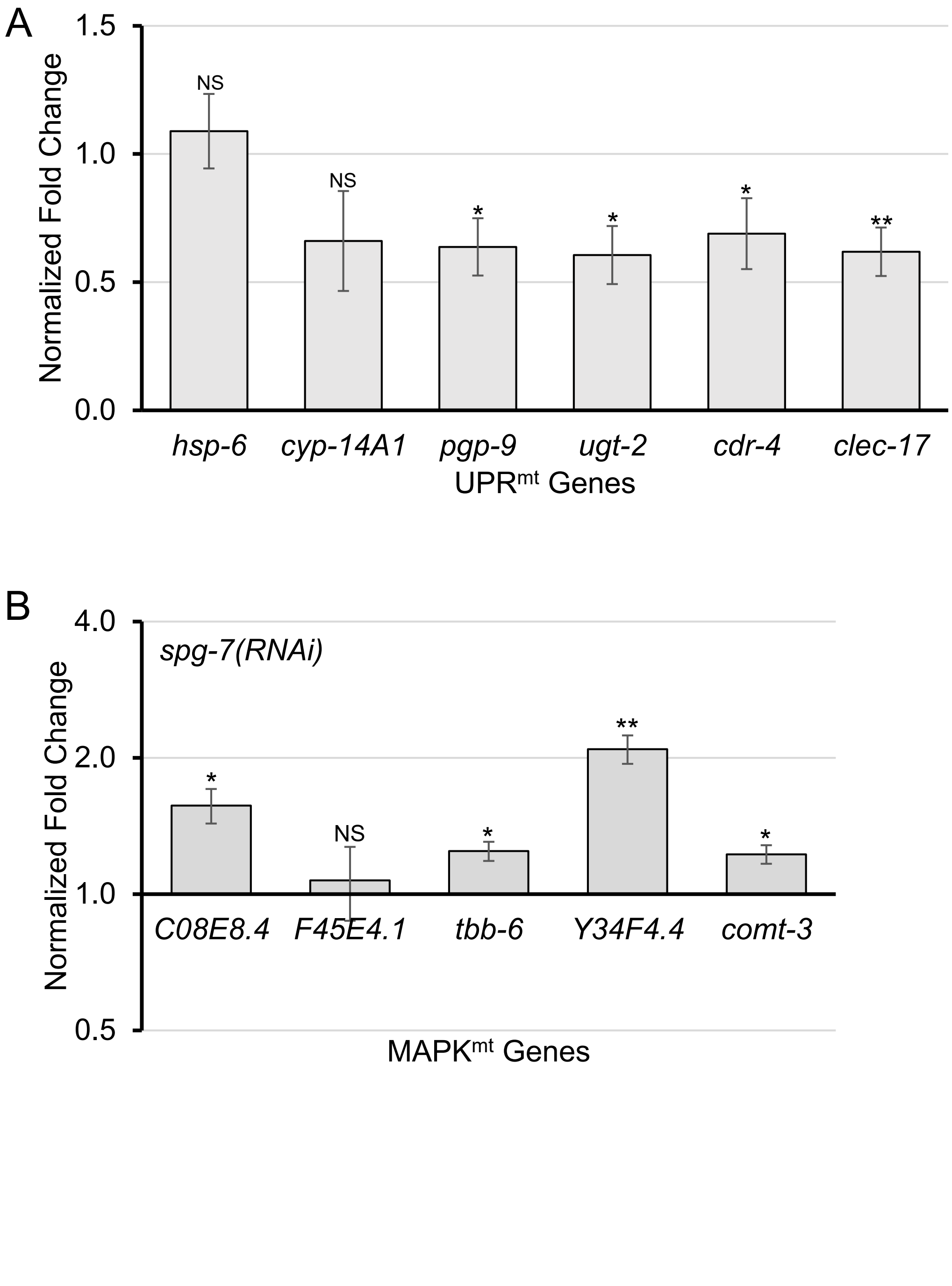
A
1.5
1.0
0.5
0.0
Normalized Fold Change
NS
NS
*
*
*
**
hsp-6
cyp-14A1
pgp-9
ugt-2
cdr-4
clec-17
UPRmt Genes
B
4.0
2.0
1.0
0.5
spg-7(RNAi)
Normalized Fold Change
*
NS
*
**
*
C08E8.4
F45E4.1
tbb-6
Y34F4.4
comt-3
MAPKmt Genes

Figure S14
