# Supplementary material for: The evolutionarily conserved ESRE stress response network is activated by ROS and mitochondrial damage
Source: BMC Biol. 2020 Jun 29;18:74. doi: 10.1186/s12915-020-00812-5 (PMC7322875; doi:10.1186/s12915-020-00812-5)
Supplement: Supplementary file 2 — Additional file 2: Table S1. Responses of mitochondrial surveillance pathway reporters to additional abiotic stressors. Summary of the activation of mitochondrial surveillance pathways after treatment with a panel of insults for 10 h, previously reported to activate ESRE. (−) indicates lack of response, plus (+), (++), or (+++) indicates weak, medium, or strong response, respectively. Three biological replicates with ~ 400 worms/replicate were analyzed. Responses were measured qualitatively. [file 12915_2020_812_MOESM2_ESM.docx]

**Table S1. Responses of mitochondrial surveillance pathway reporters to additional abiotic stressors**

| Stressors | ESRE | UPR^mt^ | MAPK |
| --- | --- | --- | --- |
| DMSO | - | - | - |
| Ethanol | + | ++ | - |
| Phenanthroline (iron chelator) | ++ | ++ | - |
| Na_2_SeO_3_ (mitophagy activator) | +++ | ++ | +++ |
| Heat shock 37°C | ++ | ++ | - |

Summary of the activation of mitochondrial surveillance pathways after treatment with a panel of insults, previously reported to activate ESRE. (-) indicates lack of response, plus (+), (++), or (+++) indicates weak, medium, or strong response, respectively. Three biological replicates with ~400 worms/replicate were analyzed. Responses were measured qualitatively.
